# Ectomesenchymal identity emerges via relief of *Twist1* transcript destabilisation

**DOI:** 10.1038/s41467-026-76557-6

**Published:** 2026-08-11

**Authors:** Lara C. Busby, Jessica R. Patrick, Luke W. Lyons, Abby J. Bergman, Megan L. Martik

**Affiliations:** 1 Department of Molecular and Cell Biology, University of California, Berkeley, Berkeley, CA USA; 2 Department of Genetics, Stanford University, Palo Alto, CA USA

**Keywords:** Differentiation, Embryology, Evolutionary developmental biology, Multipotent stem cells, Development

## Abstract

During vertebrate development, a subset of cranial neural crest cells (CNCCs) termed ‘ectomesenchyme’ differentiates into cell types canonically associated with the mesoderm (cartilage, bone and muscle). While the molecular decisions that guide CNCCs toward ectomesenchymal identity remain incompletely understood, the transcription factor *Twist1* plays a central role. Here, we show that while *Twist1* transcripts accumulate in late migratory CNCCs as cells enter the pharyngeal arch environment, a *Twist1* enhancer within *Hdac9* is active in the neural tube and CNCCs. We reconcile the temporal discrepancy between enhancer activity and transcript accumulation by showing that the *Twist1* 3’ UTR from multiple vertebrate species (but not the non-vertebrate chordate *Ciona intestinalis*) destabilises transcripts in the ectoderm via a conserved AU-Rich Element. Together, these findings reveal a vertebrate-specific, two-tiered regulatory mechanism that uncouples enhancer activity from transcript accumulation, gating the onset of *Twist1* expression and the acquisition of ectomesenchymal identity in vertebrate CNCCs.

## Abstract (web summary)

Here they identify a *Twist1* enhancer that is activated early in the migrating cranial neural crest but the *Twist1* 3' UTR contains a vertebrate-specific AU-rich element that destabilises transcripts until ventral head signals relieve the repression, thereby gating ectomesenchymal identity.

## Introduction

The evolution of the vertebrate phylum is intimately associated with the emergence of a novel embryonic cell type, the neural crest (NC), which gives rise to a remarkable variety of cell type derivatives during embryogenesis^1^. Despite their ectodermal origin in the neural plate border, neural crest cells (NCCs) give rise to both ectodermal derivatives as well as cell types canonically associated with the mesoderm, including chondrocytes, smooth muscle cells, and cardiomyocytes. These NCCs that form mesoderm-like derivatives are termed ectomesenchyme, reflecting their origin in the ectoderm and acquisition of mesenchymal characteristics^2–4^. Though initially controversial, the contribution of NCCs to mesenchymal derivatives is now widely accepted and recognised as a key evolutionary innovation that enabled the expansion of the vertebrate head and adoption of a predatory lifestyle^5^.

Seminal fate-mapping studies in a variety of vertebrate models have shown that the ability to give rise to some derivatives is restricted to subpopulations of NCCs that arise at distinct positions along the anteroposterior (AP) axis, a phenomenon termed axial regionalisation. In amniotes, only cranial neural crest cells (CNCCs), which arise anterior to rhombomere 6, give rise to chondrocytes and osteocytes that form the majority of the craniofacial skeleton, including the jaw. Classical grafting experiments demonstrated that trunk NCCs transplanted to the cranial region do not contribute to cartilage of the developing head^6–8^. However, CNCCs transplanted into the trunk region can form ectopic cartilage nodules^9^. These findings suggest that there is an intrinsic difference in the potency of NCCs along the AP axis that is established prior to their emigration from the neural tube.

More recent work has demonstrated that these differences in potency are controlled by the local activation of gene regulatory subcircuits along the AP axis. Genes encoding a suite of cranial-specific transcription factors (*Brn3c*, *Lhx5*, *Dmbx1*, *Sox8*, *Tfap2b* and *Ets1*) are expressed by premigratory CNCCs, but not by premigratory trunk neural crest cells in amniotes^10^. When three of these cranial transcription factors (*Sox8*, *Tfap2b*, and *Ets1*) were overexpressed in trunk NCCs, reprogrammed trunk NCCs were able to give rise to chondrocytes upon transplantation to the head environment, suggesting that these early cranial identity genes are sufficient to imbue trunk NCCs with ectomesenchymal potential^10^. Subsequently, this cranial-specific NC Gene Regulatory Network (GRN) was shown to have arisen progressively throughout vertebrate evolution with gradual addition of network components in gnathostome lineages^11^.

Arising in the dorsal neural tube, CNCCs fated to become ectomesenchyme migrate ventrally beneath the surface ectoderm into the pharyngeal arches, where, late in their migration, they undergo a transcriptional switch. This switch involves downregulating the expression of early migratory NC genes (such as *Sox10* and *Foxd3*) while upregulating the expression of ectomesenchymal genes (including *Dlx2*)^12^ and is dependent on arch-derived extrinsic signals, including FGF. Inhibition of FGF signalling disrupts this switch, causing CNCCs to inappropriately retain expression of *Sox10* and *Foxd3* in the pharyngeal arches^12^. Thus, previous work has hinted at key features of the intrinsic CNCC transcriptional state at premigratory stages, as well as aspects of the extrinsic signals from the head environment that are important for the emergence of ectomesenchyme. However, we currently lack a full understanding of the molecular mechanisms by which cells adopt ectomesenchymal identity.

In this study, we dissect the regulatory mechanisms that govern the acquisition of ectomesenchymal identity in CNCCs by focusing on the regulation of *Twist1*. *Twist1* has been implicated as a key node in the ectomesenchymal CNCC GRN from single-cell RNA sequencing of sorted mouse CNCCs, where it was implicated in the decision between neuronal and ectomesenchymal cell fate^13^. Overexpression of *Twist1* in trunk NCCs is sufficient to redirect the migratory path of these cells away from the dorsal root ganglion (toward the dermis) and activate the expression of *Prrx2*, another key mesenchymal identity gene^13^. Additionally, numerous experiments in multiple vertebrate species have shown that loss of *Twist1* function in CNCCs results in a failure to activate ectomesenchymal gene programmes and leads to maintained expression of early NC genes, in some cases biasing cells toward neuronal identity^14–17^. Notably, this phenocopies the consequences of loss of FGF signalling from the pharyngeal arches^12^, suggesting that *Twist1* may act upstream of other ectomesenchymal identity genes.

Here, we show that ectomesenchymal identity emerges late during the migration of CNCCs to their destinations in the ventral head. Using previously published Hi-ChIP, CUT&RUN, and ATAC-seq data from avian CNCCs, we identify a distal enhancer that contacts the *Twist1* locus and drives robust reporter gene expression in the neural tube and migratory CNCCs. Surprisingly, this enhancer is active well before endogenous accumulation of the *Twist1* transcript, suggesting that there is an additional level of regulation of *Twist1* transcripts. We resolve this paradox by showing that the *Twist1* 3′ UTR potently destabilises GFP transcripts in the neural tube and early migratory CNCCs, indicating that post-transcriptional regulation is a key contributor to the emergence of ectomesenchyme.

Together, our findings reveal a two-tiered regulatory mechanism that ensures *Twist1* transcripts accumulate only once CNCCs reach the ventral head environment. This mechanism explains how ectomesenchymal identity is precisely controlled in space and time.

## Results

### *Twist1* is expressed by late migratory ectomesenchyme

To characterise the emergence of ectomesenchymal cell states in space and time, we performed multiplexed in situ hybridisation^18^ to detect transcripts of the ectomesenchyme-associated gene *Twist1*, as well as NC specifier genes *Sox10* and *Ets1*. Co-staining for NC specifier gene expression was crucial for interpretation in these analyses because *Twist1* is broadly expressed in mesodermal tissues across vertebrates during development.

**Fig. 1:**
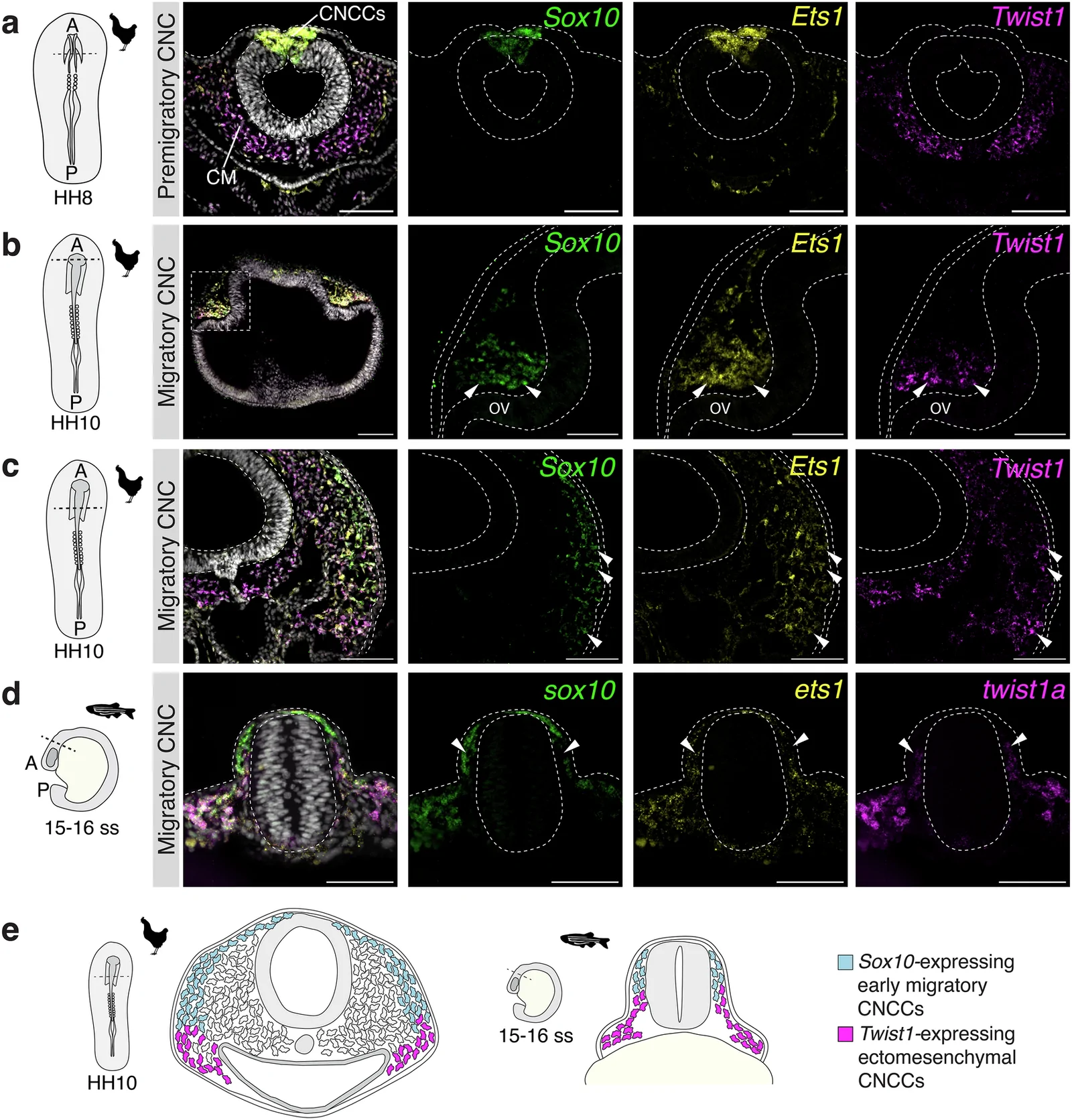
*Twist1* is expressed by late migratory ectomesenchyme. **a**–**d** Summed slice intensity projections of multiplexed HCR staining for transcripts of the early neural crest specifier gene *Sox10*, the cranial NC gene *Ets1* and *Twist1*. In each panel, the leftmost image is a composite of DAPI, *Sox10*, *Ets1* and *Twist1* transcripts, and the remaining images (left to right) are single-channel images for *Sox10*, *Ets1* and *Twist1,* respectively. **a**–**c** Staining in chicken embryo transverse sections. **a** shows that premigratory CNCCs in the dorsal neural tube at HH8 express both *Sox10* and *Ets1* but not *Twist1,* while underlying cranial mesoderm (CM) expresses *Twist1* at this stage (*n* = 5/5). Sections from HH10 chicken embryos show that *Twist1* is only upregulated in late migratory CNCCs **b** overlying the prospective optic vesicle (OV) (*n* = 5/5) and **c** more posteriorly within the cranial region in cells migrating beneath the surface ectoderm into the pharyngeal arches (*n* = 5/5). **d** Zebrafish embryo cranial region section at 15–16ss stained for *sox10*, *ets1* and *twist1a* (*n* = 3/3). **e** Schematic summarising *Twist1* expression analyses in chicken and zebrafish. Animal silhouettes are from Phylopic, scale bars in (**a**, **b**) (left panel) and (**c**) represent 100 µm, and in (**b**) (right three panels) and (**d**) represent 50 µm.

In chicken embryos, *Twist1* expression is detected in the cranial mesoderm from premigratory CNC stages (HH8), but not in premigratory CNCCs themselves, which are marked by *Sox10* and *Ets1* expression (Fig. 1a, Supplementary Fig. 1). By HH10, however, late migratory CNCCs close to their destinations begin to upregulate expression of *Twist1* (Fig. 1b, c). In the anterior mesenchyme overlying the forebrain, late migratory CNCCs in close apposition to the optic vesicle express *Twist1* (Fig. 1b). More posteriorly, *Sox10*/*Ets1*-positive CNCCs migrate ventrally beneath the surface ectoderm (Fig. 1c), with *Twist1* transcripts only accumulating in the most ventral cells, furthest along their migratory path (arrowheads in Fig. 1c). Immunostaining for TWIST1 protein confirms this expression dynamic (Supplementary Fig. 2). Thus, in chicken embryos, the key ectomesenchymal identity gene *Twist1* is not activated in CNCCs until they are late in migration.

To test whether this spatiotemporal dynamic is conserved, we stained *Danio rerio* embryos for *sox10*, *ets1* and *twist1a* transcripts at stages between the 4 somite stage (4ss) and 24 h post fertilisation (hpf). Consistent with previous reports^16,19,20^, *twist1a* transcripts become detectable in CNCCs as they migrate over the developing eye, as well as in late migratory and post-migratory CNCCs located in the pharyngeal arches (Fig. 1d, Supplementary Fig. 3).

In summary, we find that across the vertebrate phylum, representative species of multiple lineages express *Twist1* in the late migratory ectomesenchyme of the head (Fig. 1e). This expression challenges the long-standing view of *Twist1* as a driver of epithelial-to-mesenchymal transition in neural crest cells and instead establishes *Twist1* as a key player in the ectomesenchymal cell fate decision and a regulatory node worth dissecting further.

### Identification of a *Twist1* enhancer active in CNCCs

**Fig. 2:**
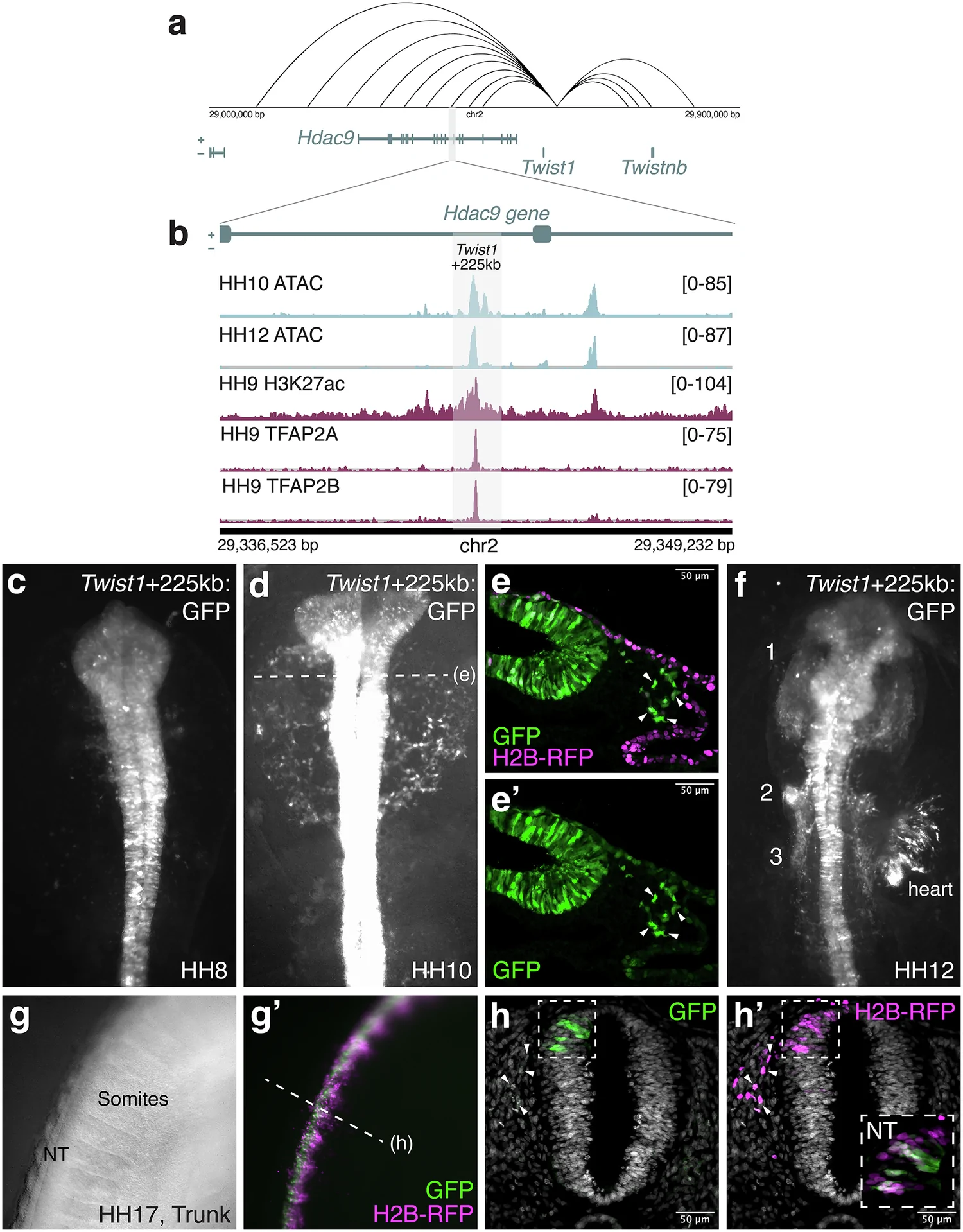
Identification of an enhancer element driving *Twist1* expression in cranial neural crest cells. **a** Contact map showing distal genomic contacts of the *Twist1* gene promoter region; the region containing *Twist1* + 225 kb is highlighted by the grey box. Raw data derived from Azambuja & Simoes-Costa (2021). **b** Multimodal tracks showing chromatin accessibility^23^, H3K27 acetylation^22^, and TFAP2A/2B genome occupancy^24^ in the region of the *Twist1* + 225 kb enhancer, located within an intron of the *HDAC9* gene. **c**, **d**, **f** GFP fluorescence driven by the *Twist1* + 225 kb: GFP enhancer reporter at HH8 (*n* = 5/5), HH10 (*n* = 12/14) and HH12 (*n* = 3/3), respectively. Numbers in (**f**) represent the three streams of cranial neural crest. **e**, **e**′ Representative transverse section showing *Twist1* + 225 kb: GFP fluorescence and H2B-RFP (electroporation control) at HH10 (*n* = 8/8 sections across 2 independent embryos). Arrowheads indicate migrating CNCCs with enhancer activity. **g**, **g**′ *In ovo* electroporations of the *Twist1* + 225 kb: GFP enhancer reporter injected into the neural tube at HH10-12 (*n* = 3/3). (**g**) shows a brightfield image of the embryonic trunk at HH17 and (**g**′) shows the corresponding composite image for GFP and H2B-RFP fluorescence. **h**, **h**′ Trunk section of *in ovo* electroporated embryo shows that GFP expression is driven in the neural tube by *Twist1* + 225 kb but despite labelling of trunk NCCs emigrating from the tube (arrowheads in **h**′) no GFP activity is present in these cells (arrowheads in **h**), suggesting that activity of the *Twist1* + 225 kb enhancer is cranial-specific in NCCs (*n* = 9/9 sections across 3 independent embryos).

To ask how *Twist1* gene expression is initiated in ectomesenchymal NC, we combined sequence conservation analysis with existing DNA-DNA contact (H3K27ac Hi-ChIP), chromatin accessibility (ATAC-seq) and Cut & Run (TFAP2A, TFAP2B, H3K27ac) datasets from sorted avian CNCCs to identify putative enhancer sequences^21–24^. We find that the *Twist1* promoter region forms numerous distal genomic contacts in dorsal neural fold cells, many within the neighbouring *Hdac9* locus (Fig. 2a, Supplementary Data 1), though none of these loops are differentially enriched in CNCCs relative to whole embryo samples. Altogether, we used enhancer reporter assays to test a total of eleven putative enhancer sequences (Supplementary Data 2) and found two sequences with mesodermal activity (Supplementary Fig. 4) and one sequence that drove activity in the neural crest (Fig. 2).

We identified a 709 bp sequence within intron 13 of the *Histone Deacetylase 9* (*Hdac9*) gene that drives robust reporter expression in the neural tube and in migrating cranial and vagal NC, herein termed *Twist1* + 225 kb (Fig. 2b–f). This genomic sequence is accessible, acetylated, and directly bound by the transcription factors TFAP2A and TFAP2B in premigratory CNCCs, supporting its role as an enhancer *(*Fig. 2b). Using an enhancer reporter assay, we found that *Twist1* + 225 kb drives expression of GFP throughout the neural plate as early as HH5 (Supplementary Fig. 5), and in the neural tube and emigrating CNCCs at HH8-HH10 (Fig. 2c–e). By HH12, *Twist1* + 225 kb labels migratory CNCCs in the three primary CNCC streams^25^ (Fig. 2f).

Given the cranial restriction of *Twist1* expression in neural crest cells, we asked whether *Twist1* + 225 kb activity was similarly restricted. Interestingly, we find that *Twist1* + 225 kb drives reporter expression in trunk neural tube cells but not in migrating TNCCs (Fig. 2g, h). Therefore, the activity of this enhancer recapitulates the cranial restriction of *Twist1* expression and ectomesenchyme emergence to the cranial region. Additionally, we have found that the chicken *Twist1* + 225 kb enhancer drives equivalent neural tube and CNCC fluorescence in zebrafish (Supplementary Fig. 6).

Taken together, our results thus far uncover a distal enhancer that contacts the *Twist1* promoter and is active in premigratory CNCCs, well before *Twist1* transcripts accumulate (Fig. 1). This striking discrepancy between enhancer activity and mRNA accumulation suggests an additional regulatory layer to precisely control *Twist1* presence and the emergence of ectomesenchymal identity. We therefore asked what upstream factors could be controlling the activity of this enhancer and whether their activity could account for the disconnect between enhancer activation and *Twist1* transcript abundance.

### *Twist1* + 225 kb is regulated by NC transcription factors

**Fig. 3:**
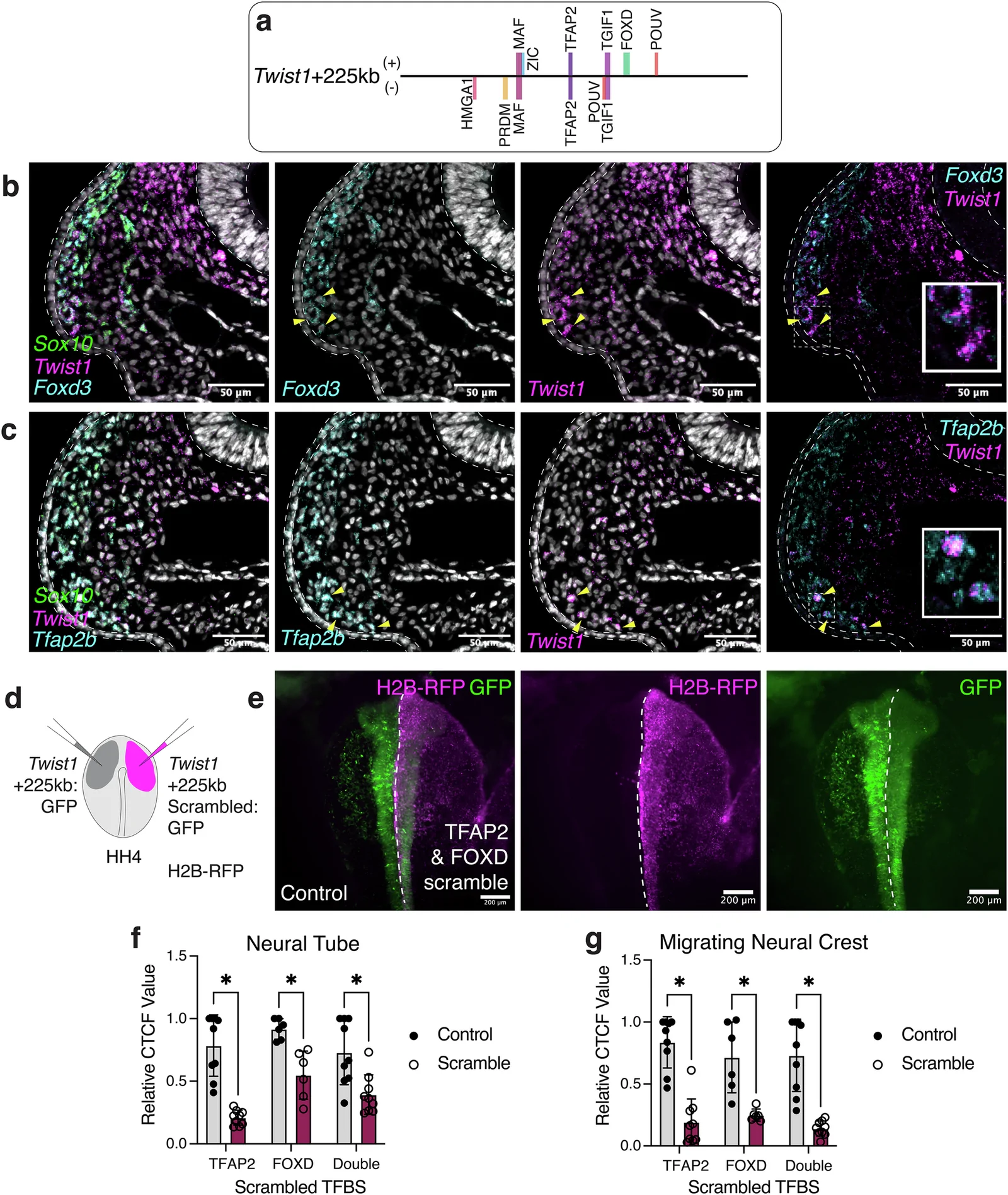
The *Twist1* + 225 kb enhancer is regulated by canonical neural crest transcription factors. **a** Box diagram of the *Twist1* + 225 kb enhancer sequence with key predicted transcription factor binding sites annotated to scale. **b** Multiplexed HCR staining for *Foxd3* and *Twist1* at HH10 shows co-expression of these two transcripts in late migratory CNCCs (*n* = 6/6 sections across 2 embryos). Yellow arrowheads indicate cells co-expressing *Foxd3* and *Twist1*. **c** Multiplexed HCR staining for *Tfap2b* and *Twist1* at HH10 shows co-expression of these two transcripts in late migratory CNCCs (*n* = 6/6 sections across 2 embryos). Yellow arrowheads indicate cells co-expressing *Tfap2b* and *Twist1*. **d** Schematic of scrambled enhancer experiment. **e** The *Twist1* + 225 kb: GFP scrambled construct shown has both TFAP2 and FOXD binding sites scrambled. Left: composite GFP and RFP fluorescence image of a representative injected embryo at HH10. Centre: H2B-RFP fluorescence (electroporation control). Right: GFP fluorescence. Quantification of GFP fluorescence intensity (corrected total cell fluorescence, CTCF) in regions of the neural tube (**f**) or lateral to the neural tube (**g**) reveals significantly reduced fluorescence resulting from scrambling of TFAP2, FOXD or TFAP2 & FOXD motifs in the *Twist1* + 225 kb enhancer sequence, suggesting that these motifs are important for enhancer activity. Statistics were calculated using two-sided unpaired *t*-tests, with all *p*-values < 0.005 (*n* = 48 measurements across 8 embryos). Exact *p*-values were: Neural Tube TFAP2 0.000004, Neural Tube FOXD 0.001505, Neural Tube Double 0.004071, Neural Crest TFAP2 0.000003, Neural Crest FOXD 0.002773, Neural Crest Double 0.000021. Bars represent the mean, and error bars represent the standard deviation. Source data are provided in the Source Data file.

To identify upstream transcription factors that regulate the *Twist1* + 225 kb enhancer, we used FIMO^26^ to predict transcription factor binding sites, which revealed the presence of putative binding sites for TFAP2, FOXD, TGIF1, MAF, ZIC, PRDM, and POUV/OCT4 (Fig. 3a). Each of these transcription factors has previously been implicated in neural crest development. FOXD3 and TFAP2A are pan-NC transcription factors that are expressed in premigratory and migratory NCCs at all axial levels, and TFAP2B is a cranial-specific transcription factor^10,24^. TFAP2A and TFAP2B have been shown to heterodimerise in CNCCs and act as pioneer transcription factors^24^. TGIF1, MAFA, and MAFB have been associated with the cardiac NC^27,28^. HMGA1 is a chromatin remodeller active in the ectoderm, including CNCCs^29^. POUV/OCT4 has been implicated in the ectomesenchymal potential of CNCCs and is maintained or reactivated in the ectoderm^30–32^. ZIC transcription factors are expressed throughout the early neural plate and subsequently in the dorsal neural tube^33^.

To test whether the spatiotemporal expression of these putative regulators is consistent with their regulation of *Twist1*, we performed HCR against *Sox10*, *Twist1* and either *Tfap2b* or *Foxd3*. We observed the co-expression of both *Tfap2b* and *Foxd3* transcripts with *Twist1* in ventral late migratory CNCCs at HH10 (Fig. 3b, c), suggesting that these transcription factors could have a functional role in driving *Twist1* expression in CNCCs. Notably, *Tfap2b* and *Foxd3* transcription factors begin to be expressed when neural crest cells are premigratory in the dorsal neural tube, much earlier than *Twist1* transcripts become detectable, suggesting they could prime *Twist1* activation via the *Twist1* + 225 kb enhancer before transcripts accumulate.

To directly test the functional importance of a subset of these binding sites in driving *Twist1* + 225 kb enhancer activity, we generated modified enhancer constructs, each with the predicted binding motif for a transcription factor scrambled. To allow comparison of relative enhancer activity after scrambling binding sites, we contralaterally electroporated the control enhancer construct and the scrambled construct with a H2B-RFP-encoding plasmid (Fig. 3d) and then assessed GFP fluorescence at HH10 (Fig. 3e). We found that constructs with a single transcription factor binding site scrambled, either TFAP2 or FOXD, led to significantly reduced activity of the enhancer in migratory CNCCs and the neural tube (Fig. 3f, g). Strikingly, scrambling both sites abolished enhancer activity in the migrating CNCCs relative to the control construct (Fig. 3e, g). Together, these results suggest that the binding of TFAP2 and FOXD transcription factors to the *Twist1* + 225 kb sequence is essential for enhancer activity in neural crest cells.

Despite robust enhancer activation via binding of canonical neural crest transcription factors, *Twist1* transcripts do not accumulate until much later in migration. This paradox, therefore, challenges the conventional assumption that enhancer activity and transcript accumulation are tightly coupled. To resolve this, we considered that additional layers of regulation may gate the precise timing of *Twist1* expression and therefore the emergence of ectomesenchymal identity.

### The *Twist1* transcript is post-transcriptionally destabilised

**Fig. 4:**
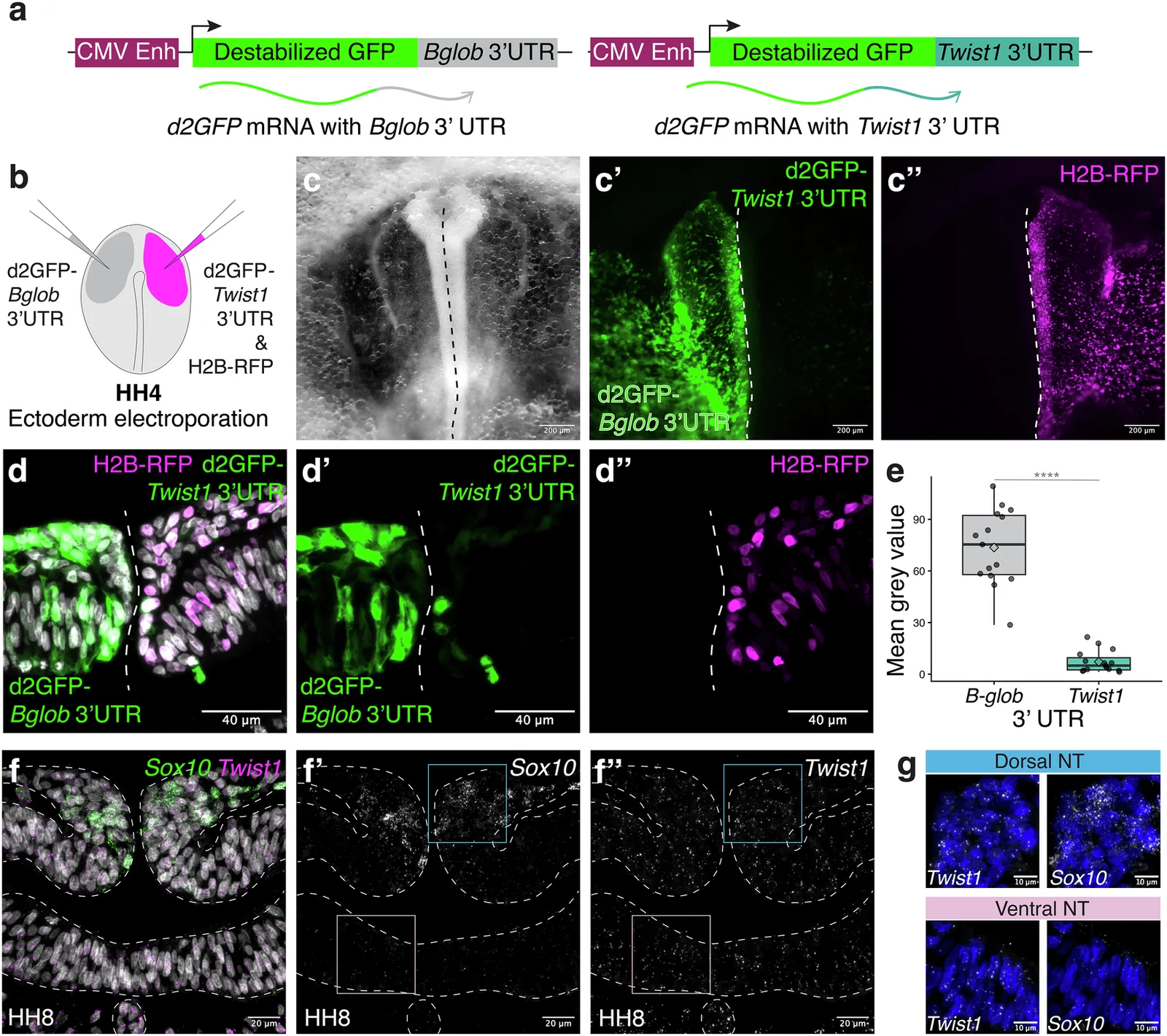
The 3′ UTR of *Twist1* is sufficient to destabilise transcripts in the ectoderm. **a** Schematic of constructs used to test post-transcriptional regulation. **b** Schematic representation of the electroporation schema to test for post-transcriptional regulation of *Twist1* transcripts. The *β-globin* 3′ UTR reporter was electroporated on the left side of the embryo, and the *Twist1* 3′ UTR reporter (with H2B-RFP plasmid as control) was electroporated on the right side of the embryo. **c** Single-channel images of a representative embryo at HH8-9 showing (**c**) brightfield overview, (**c**′) GFP channel signal and (**c**′′) RFP channel signal (*n* = 15/15). The dashed line denotes the midline of the embryo. **d** Representative section of the cranial region at HH9 showing (**d**) composite image with DAPI, GFP and RFP signal, (**d**′) the GFP single-channel image and (**d**′′) the RFP single-channel image. **e** Quantification of GFP signal intensity in segmented electroporated cells on either side of the embryo (*n* = 15 sections across 3 embryos). Statistics calculated using a two-sided paired *t*-test, *p* < 0.0001. Box plot shows the median (centre line), 25th and 75th percentiles (box limits), whiskers extend to the maximum and minimum observations within 1.5× the interquartile range, and the mean for each sample as a diamond. Source data are provided in the Source Data file. **f** Representative cranial transverse section at HH8 showing the neural tube stained for *Sox10* and *Twist1* transcripts. While *Sox10* transcripts are concentrated in the dorsal neural tube (**f**′), nuclear *Twist1* transcript puncta are detectable throughout the neural tube (**f**′′) (*n* = 9/9 sections across 3 embryos). **g** Composite DAPI and single HCR channel images for the inset boxes placed on the dorsal and ventral regions of the neural tube (NT) (note that a similar result was obtained in *n* = 9/9 sections across 3 embryos).

Given the clear difference in timing between the onset of *Twist1* + 225 kb enhancer activity and the accumulation of *Twist1* transcripts (late migratory CNCCs), we asked whether *Twist1* transcripts may be subject to post-transcriptional regulation. Post-transcriptional regulation of a transcript commonly occurs via sequence and/ or structural elements found in the 3′ untranslated region (3′ UTR). To test the influence of the *Twist1* 3′ UTR on transcript stability, we cloned a construct consisting of a ubiquitous promoter driving the expression of destabilised GFP followed by the chicken *Twist1* 3′ UTR (Fig. 4a). As a control, we electroporated an equivalent construct with the *β-globin* 3′ UTR (Fig. 4a), which is known to stabilise transcripts in a variety of cellular contexts^34,35^. To enable direct side-by-side comparison of GFP fluorescence as a readout of transcript stability, we electroporated the *Twist1* 3′ UTR construct (with a H2B-RFP plasmid as an electroporation control) on the right side of the embryo, and the *β-globin* 3′ UTR construct on the left side (Fig. 4a, b). We observed bright GFP fluorescence in the neural tube and surface ectoderm on the left side of the HH8-10 embryo but little or no fluorescence in the same tissues on the right, *Twist1* 3′ UTR side of the embryo (Fig. 4c–e). This result demonstrates that the *Twist1* 3′ UTR is strongly post-transcriptionally regulated in ectodermal tissues at this stage, effectively preventing *Twist1* transcript and protein accumulation despite an active enhancer.

Given this strong post-transcriptional destabilisation, we next asked whether *Twist1* transcripts are detectable in cells that exhibit both enhancer activity and transcript destabilisation (neural tube and early migratory CNCCs). We stained HH8-10 embryos for *Sox10* and *Twist1* transcripts and imaged them at higher magnification. This imaging revealed the presence of nuclear puncta of *Twist1* transcripts throughout the neural tube, whereas *Sox10* transcript puncta are concentrated in the dorsal neural tube, as expected (Fig. 4f, g). These widespread *Twist1* transcript puncta coincide with the activity of the *Twist1* + 225 kb enhancer (Fig. 2c–e) and confirm that *Twist1* is indeed transcribed throughout the neural tube. Nonetheless, TWIST1 protein is undetectable in the neural tube and in CNCCs until they are late in their migration (Supplementary Fig. 2). These results suggest that *Twist1* transcripts are produced in the neural tube and early CNCCs, but destabilised via their 3′ UTR, preventing protein production and acquisition of ectomesenchymal identity. Similarly, we have also found that in mouse embryos, TWIST1 protein is not detectable in premigratory or early migratory CNCCs, despite detectable *Twist1* transcripts (Supplementary Fig. 7), consistent with previous observations^36^.

### *Twist1* destabilisation protects the neuroepithelium

**Fig. 5:**
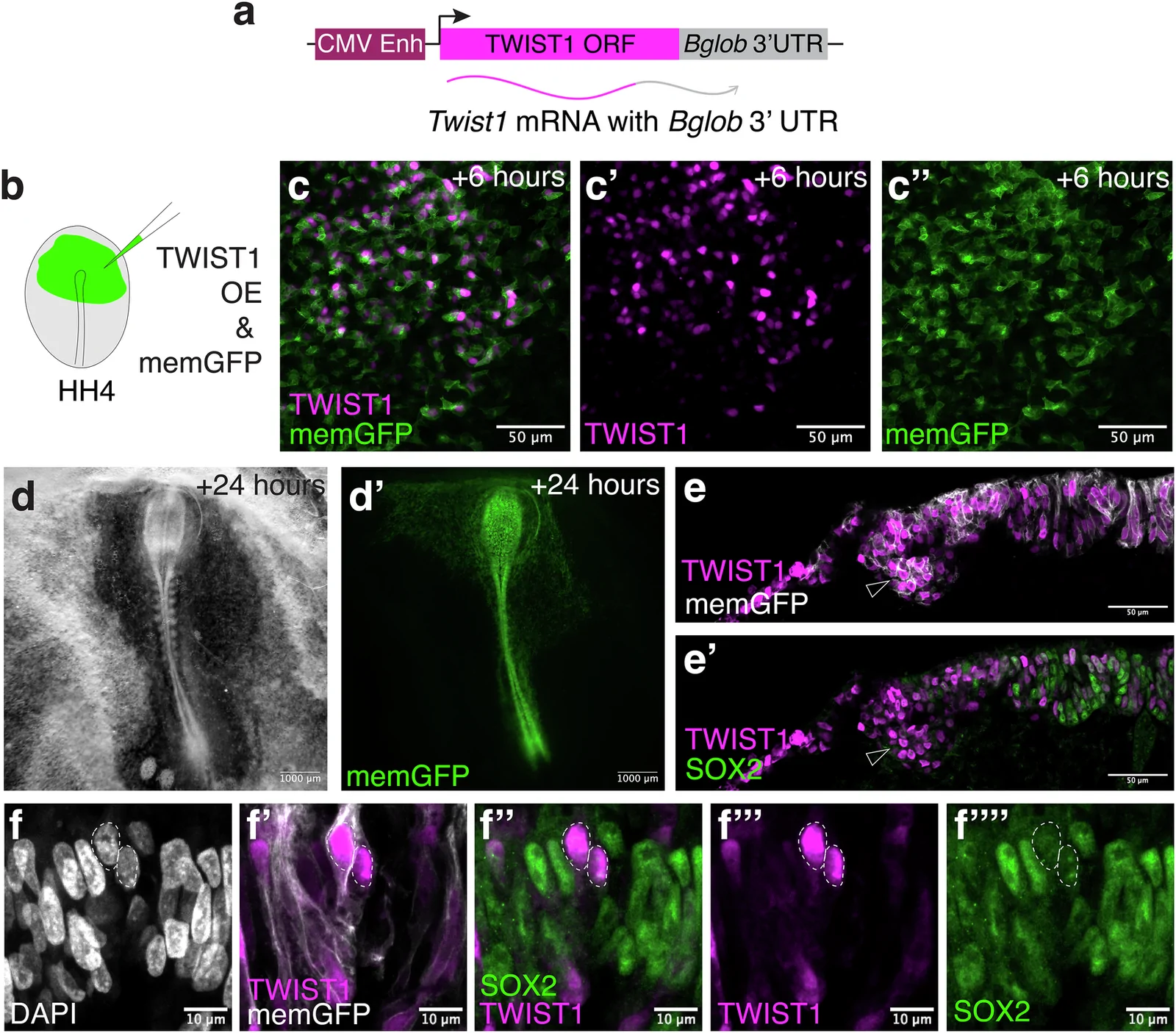
Expression of stabilised *Twist1* transcripts in the neural plate results in cranial neural tube closure defects. **a** Schematic of the construct used to over-express TWIST1 in neural plate cells. A codon-optimised TWIST1 ORF is followed by the *β-globin* 3′ UTR, allowing the production of highly stable *Twist1* transcripts in the neural plate. **b** Schematic showing the electroporation schema used. The TWIST1 OE construct was co-electroporated with a construct encoding a membrane-targeted GFP. **c** Whole-mount fluorescence images of the neural plate 6 h after electroporation showing mosaic expression of membrane GFP and nuclear TWIST1 protein (*n* = 6/6 embryos). **c** is the composite image, **c**′ is the TWIST1 single-channel image and **c**′′ is the GFP single-channel image. **d** At 24 h after electroporation, many HH10 embryos (*n* = 3/6) exhibited significant neural tube defects with an open neural plate in the cranial region. A representative embryo is shown in brightfield (**d**) and GFP (**d**′) channels. **e** Section through the cranial region showing TWIST1-expressing cells dispersed throughout the SOX2-expressing neural plate. The section is centred on the lateral region of the neural plate with the midline at the right side of the image. **e** Composite membrane GFP and TWIST1 image, **e**′ composite TWIST1 and SOX2 image. Open arrowheads indicate the position of ingressing cells at the neural plate border (*n* = 3/3 embryos). **f** Summed slice projection showing an example of reduced nuclear SOX2 in cells overexpressing TWIST1. **f** single-channel DAPI, **f**′ composite membrane GFP and TWIST1 image, **f**′′ composite TWIST1 and SOX2 image, **f**′′′ single-channel TWIST1 and **f**′′′′ single-channel SOX2 image. The white dotted lines indicate the positions of two nuclei with strong TWIST1 protein enrichment. Note that a similar result was obtained in *n* = 6/6 sections across 3 embryos.

To ask what the functional significance of *Twist1* destabilisation in the neural tube is, we overexpressed *Twist1* (with the *β-globin* 3′ UTR) in the neural plate from HH4-5 (Fig. 5a, b). Within 6 h of electroporation, TWIST1 protein is detectable in electroporated cells of the neural plate (marked by co-electroporation with a membrane GFP-encoding plasmid), but not in those neighbouring cells that have not taken up the plasmid (Fig. 5c). At HH8-12, many electroporated embryos exhibited severe neural tube closure defects, with an open neural plate in the cranial region, but morphologically normal neural tubes in the trunk region (Fig. 5d). In section, the neural plate appeared disorganised, but cells could still be identified ingressing from the epithelium (arrowheads in Fig. 5e), suggesting that neural crest cells are still specified in the event of precocious TWIST1 expression. We hypothesised that the overexpression of TWIST1 alters the cellular state of neural progenitors. To test this, we stained sections for SOX2 protein, an essential transcription factor required to maintain the proliferative capacity of neural progenitor cells and prevent their premature differentiation^37^. We found that many cells with nuclear TWIST1 showed lower or no enrichment of SOX2 protein (example shown in Fig. 5f), suggesting that expression of TWIST1 compromises neural progenitor identity. Together, these results suggest that *Twist1* transcript destabilisation allows for early transcription while (1) protecting the neuroepithelium from the adverse effects of TWIST1 protein expression, and (2) controlling the spatiotemporal context of ectomesenchyme emergence.

Therefore, we next sought to understand the precise mechanism by which the post-transcriptional repression is relieved to permit *Twist1* transcript abundance and acquisition of ectomesenchymal identity.

### *Twist1* transcript stabilisation requires spatial cues

**Fig. 6:**
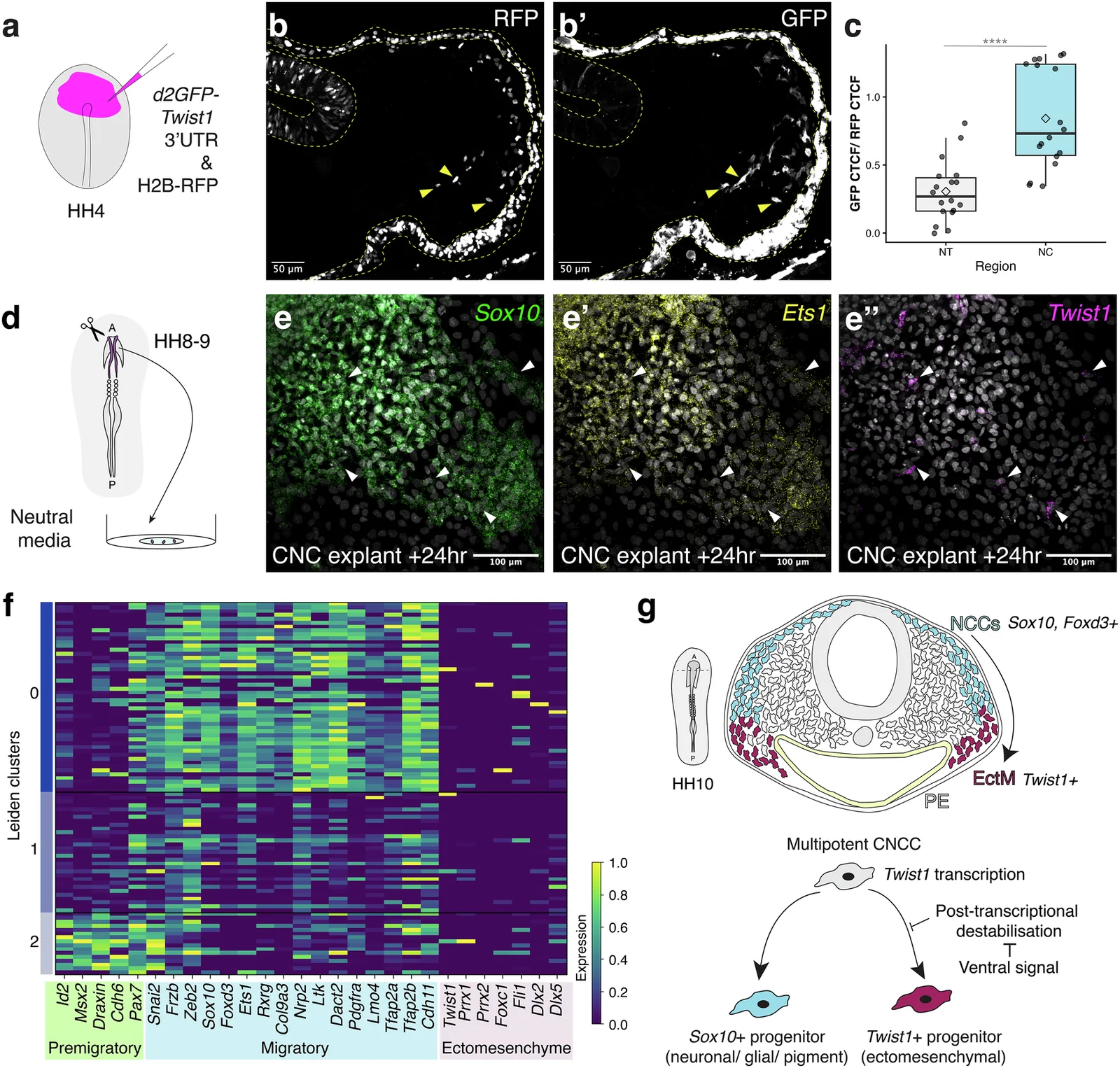
The post-transcriptional destabilisation of *Twist1* transcripts is relieved by extrinsic signals to control the context of ectomesenchyme differentiation. **a** Schematic representation of ectoderm electroporation. Single-channel images of a representative HH10 embryo in section with RFP signal in (**b**) and GFP signal in (**b**′) (*n* = 3/3 embryos). The GFP signal was amplified by antibody staining. Yellow arrowheads indicate positions of migrating neural crest cells. **c** Quantification of GFP-corrected total cell fluorescence (CTCF) normalised to RFP CTCF in regions encompassing neural tube (NT) and migrating neural crest (NC) cells (*n* = 18 (3 measurements per section, 3 sections per embryo, 2 embryos)). Significance calculated using two-sided Welch’s *t*-test, *p* = 0.00001372. Box plot shows the median (centre line), 25th and 75th percentiles (box limits), whiskers extend to the maximum and minimum observations within 1.5× the interquartile range, and the mean for each sample as a diamond. Source data are provided in the Source Data file. **d** Schematic of cranial neural crest explant – dorsal neural folds were dissected from HH8-9 embryos and explanted on fibronectin-coated glass dishes. **e** Multiplexed HCR staining for *Sox10* (**e**), *Ets1* (**e**′) and *Twist1* (**e**′′) transcripts in CNC explants fixed after 24 h of culture. Arrowheads indicate the few Twist1-expressing cells in the explant (*n* = 3/3). **f** Heatmap summarising relative expression of key premigratory CNCC, migratory CNCC and ectomesenchymal gene expression markers in explanted CNCCs at 24 h of culture. Raw data were obtained from ref. ^38^. Explanted CNCCs in culture express known markers of in vivo migratory CNCCs but lack expression of ectomesenchymal markers. **g** Schematic summarising model for the emergence of ectomesenchymal identity in CNCCs. NCCs neural crest cells, EctM ectomesenchyme.

When the *Twist1* 3′ UTR reporter construct was electroporated throughout the epiblast and primitive streak of the embryo (Supplementary Fig. 8a), we observed bright GFP fluorescence within the extra-embryonic tissue (Supplementary Fig. 8c) as well as the somites (Supplementary Fig. 8d). These results suggest that the destabilisation of the *Twist1* transcript specifically occurs in ectodermal tissues. For ectomesenchymal identity to emerge in post-migratory CNCCs, we reasoned that this destabilisation would need to be lifted to allow *Twist1* transcript and protein to accumulate. Thus, we next asked whether we could detect higher *Twist1* UTR reporter fluorescence in migrating CNCCs relative to neural tube cells. In sectioned *Twist1* 3′ UTR reporter embryos stained for GFP protein, we observed significantly higher GFP fluorescence in late migratory CNCCs positioned ventrally within the head relative to electroporated cells within the neural tube, suggesting that the intrinsic stability of *Twist1* transcripts is modified as CNCCs leave the neural tube and migrate ventrally (Fig. 6a–c).

One possible explanation for the higher *Twist1* 3′ UTR reporter fluorescence in CNCCs as they migrate ventrally is that the extrinsic signalling environment of the ventral head influences transcript stability. To test whether extrinsic signals are required for *Twist1* transcript stabilisation, we used an explant approach to assay neural crest gene expression when cultured in neutral media. Cranial neural folds were dissected from HH8-9 embryos, explanted on fibronectin in neutral media, and cultured for 24 h (Fig. 6d). In contrast to in vivo development, where these cells normally would accumulate *Twist1* transcripts, explants stained strongly for *Sox10* and *Ets1* but not *Twist1* (Fig. 6e), suggesting that extrinsic cues from the embryonic environment are required for *Twist1* transcript stabilisation. Additionally, we analysed previously published single-cell RNA-sequencing data from explanted avian CNCCs at 24 h in culture^38^, and found that, consistent with our staining data, while these cells express numerous previously described markers^39^ of early migratory CNCCs, they do not express ectomesenchymal identity genes (Fig. 6f). These results suggest that CNCCs outside of the embryonic environment resemble migratory CNCCs in vivo. In the absence of extrinsic signalling inputs, they do not undergo the cell state transition to ectomesenchymal progenitor, suggesting that inputs from surrounding tissues are required to alleviate *Twist1* transcript destabilisation.

Together, our observations support a model (Fig. 6g) where *Twist1* transcription is activated in premigratory CNCCs within the neural tube, but *Twist1* transcripts are highly unstable in the neural tube and dorsal head environment. As CNCCs migrate ventrally into the pharyngeal arches, they receive extrinsic signals that lead to the stabilisation of *Twist1* transcripts, allowing rapid upregulation of *Twist1* and establishment of ectomesenchymal identity. Our model, decoupling enhancer priming and transcript stabilisation, would ensure that ectomesenchymal commitment is acquired in the correct anatomical context and protect the neuroepithelium from the adverse consequences of TWIST1 activity. Such a mechanism may represent an evolutionary innovation that allowed vertebrates to precisely coordinate the spatial context of neural crest differentiation into ectomesenchyme. To test whether this regulatory logic is conserved across species, we next turned to interspecies UTR experiments.

### *Twist1* destabilisation is conserved across vertebrates

**Fig. 7:**
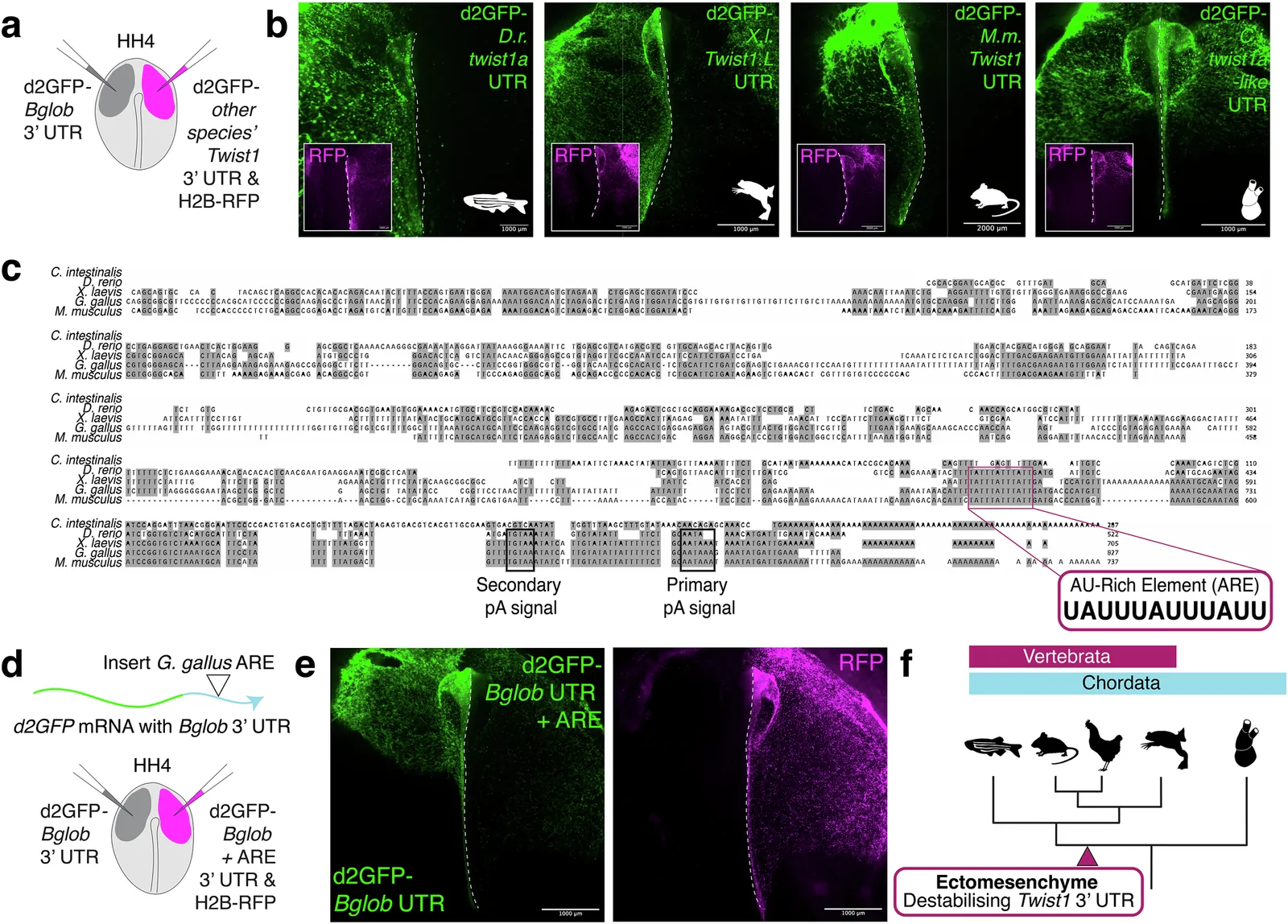
Post-transcriptional destabilisation of *Twist1* transcripts is vertebrate-specific. **a** Schematic representing the electroporation schema for interspecies UTR reporter experiments. The *β-globin* 3′ UTR reporter was electroporated on the left side of the embryo, and the *Twist1* 3′ UTR reporter from a different species was electroporated on the right side of the embryo with the H2B-RFP electroporation control. **b** GFP fluorescence images from interspecies UTR reporter experiments (left-right: *Danio rerio twist1a* (*n* = 8/8), *Xenopus laevis Twist1.L* (*n* = 8/8), *Mus musculus Twist1* (*n* = 5/5) and *Ciona intestinalis twist1a-like* (*n* = 10/10)). The dotted white line denotes the midline of the embryo. Inset images in (**b**) are the corresponding RFP fluorescence images. **c** Multiple sequence alignment of (top–bottom) *C. intestinalis twist1a-like*, *Danio rerio twist1a*, *Xenopus laevis Twist1.L*, *Gallus gallus Twist1* and *Mus musculus Twist1* 3′ UTR sequences. Empty positions denote gaps in the alignment; shaded nucleotides are conserved across three or more sequences. Annotation shows a conserved AU-Rich Element (ARE) that is present across all four vertebrate sequences but absent in the *C. intestinalis* sequence. **d** Schematic of *β-globin* UTR + ARE experiment. The *G. gallus* ARE was introduced into the *β-globin* 3′ UTR reporter, then this modified UTR reporter construct was electroporated on the right side of the embryo with the unmodified control *β-globin* 3′ UTR reporter on the left side of the embryo. **e** Representative fluorescence images of UTR reporter experiment (*n* = 13/13). **f** Schematic chordate phylogeny. Novel destabilising elements in the *Twist1* 3′ UTR evolved in vertebrates and are not present in their closest non-vertebrate chordate relatives.

To ask whether this phenomenon is species-specific, or if the 3′ UTRs from *Twist1* orthologues across vertebrates show the same destabilising influence, we cloned additional constructs consisting of the d2GFP open reading frame followed by either the zebrafish (*Danio rerio*) *twist1a* 3′ UTR, the mouse (*Mus musculus*) *Twist1* 3′ UTR, or the frog (*Xenopus laevis*) *Twist1.L* 3′ UTR. All of these constructs produced a very similar result to the chicken construct upon electroporation into the chicken epiblast (Fig. 7a, b), suggesting that the sequence elements responsible for destabilisation of *Twist1* transcripts in the ectoderm are conserved. We also produced an equivalent construct containing the *twist1a-like* 3′ UTR from the non-vertebrate chordate *Ciona intestinalis*. Interestingly, this construct led to bright GFP fluorescence in the neural tube and surface ectoderm (Fig. 7b), indicating that ectodermal *Twist1* destabilisation is absent and that sequence elements contributing to transcript destabilisation evolved in vertebrates.

### A conserved AU-rich element confers destabilisation

Given the high degree of functional conservation that we observed in our interspecies 3′ UTR reporter experiments, we next asked whether there are conserved sequence elements across vertebrate *Twist1* 3′ UTRs and absent from non-vertebrate *Twist* 3′ UTRs. We performed a multiple sequence alignment of *Twist1* 3′ UTR sequences from *C. intestinalis, D. rerio, X. laevis, G. gallus* and *M. musculus* (Fig. 7c), finding that the most highly conserved sequence elements present in all four vertebrate species were concentrated toward the 3′ end of these UTR sequences. In particular, we noted the presence of an AU-Rich Element (ARE), UAUUUAUUUAUU, that is conserved across all four vertebrates but absent from the *C. intestinalis* 3′ UTR (Fig. 7c). This ARE contains the nonamer, UUAUUUAUU, which has been previously shown to be the key sequence determinant mediating mRNA deadenylation and degradation^40,41^.

To ask whether this ARE is sufficient to destabilise an otherwise stable transcript in the neural tube and surface ectoderm, we inserted a 30nt fragment of the *G. gallus Twist1* 3′ UTR encompassing the conserved ARE into the *β-globin* 3′ UTR construct (Fig. 7d). When we electroporated this *β-globin* + ARE UTR construct, we found that it closely recapitulated the minimal fluorescence observed in the *Twist1* 3′ UTR experiments, confirming that this ARE potently destabilises transcripts in the ectoderm and can account for the destabilising effect of vertebrate *Twist1* 3′ UTRs (Fig. 7e). Together, our data demonstrate that post-transcriptional repression is an important contributor to ectomesenchymal fate selection across vertebrates (Fig. 7f), and evolution of the neural crest is associated with acquisition of a novel destabilising ARE in the *Twist1* 3′ UTR.

## Discussion

### *Twist1* is an ectomesenchymal identity gene (not an EMT driver) in neural crest cells

*Twist* genes, which encode bHLH transcription factors, were first described in *Drosophila melanogaster* as key regulators of mesodermal identity and epithelial-to-mesenchymal transition (EMT) in ventral furrow cells^42^. Subsequently, the role of *Twist* genes in mesodermal identity was found to be widely conserved across metazoans, including the vertebrate lineage^43^. Additionally, when *Twist1* was cloned in *Xenopus*, it was found to be expressed in CNCCs from neural plate border stages (stage 14.5+) and was subsequently implicated in the EMT of these cells^44,45^. This led to the conception of *Twist1* as a key driver of EMT in neural crest cells, and consequently *Twist1* has been frequently included in Gene Regulatory Networks (GRNs) for neural crest cell specification at the neural plate border^46–48^.

Our data challenges this long-standing paradigm. In this study, we show that *Twist1* transcripts do not accumulate in CNCCs until they are late in their migration, a timing that is inconsistent with a role for *Twist1* in driving neural crest EMT. Consistent with this, global and tissue-specific *Twist1* mouse mutants do not disrupt neural crest delamination or migration into the frontonasal prominence and pharyngeal arches; instead, these CNCCs fail to acquire ectomesenchymal identity, maintaining inappropriate expression of *Sox10* in the pharyngeal arches and frontonasal prominence^14,49^. A very similar phenotype is observed in *twist1a/1b* double morphants or mutants in zebrafish^16,50^. Thus, *Twist1* is dispensable for EMT but essential for CNCC commitment to ectomesenchymal derivatives.

Interestingly, parallels exist in the context of *Drosophila* mesoderm development, where it has been shown that initiation of invagination and mesodermal identity emergence can be uncoupled. In the absence of *twist*, *snail* can drive invagination of prospective mesoderm cells, but these cells fail to upregulate mesodermal markers such as *tinman* (*Nkx2-5*) and *bagpipe* (*Bapx1*)^51^. This result reinforces the idea that *Twist* genes in the mesoderm act primarily to reinforce cellular identity, consistent with our observations in neural crest cells.

Together, our results reframe our conception of *Twist1* in neural crest biology. Rather than driving EMT, *Twist1* functions primarily as a gatekeeper for acquiring ectomesenchymal identity. Our findings not only resolve long-standing inconsistencies in the literature about the role of *Twist1* in early neural crest development but also highlight *Twist1* as a critical node in the regulatory circuitry that enabled ectoderm-derived neural crest cells to generate mesenchymal derivatives.

### Cell-intrinsic and extrinsic contributors to the ectomesenchymal cell fate decision

As discussed in the introduction, cranial neural crest cells express a distinct suite of transcription factors that have been shown to be sufficient to imbue trunk neural crest cells with the potency to form cartilage, an ectomesenchymal derivative^10^. Additionally, extrinsic signalling cues from the head environment have been implicated in the emergence of ectomesenchymal identity and chondrocyte differentiation. For example, when quail CNCCs are dissociated at HH9 and cultured as isolated clones, they differentiate into glia, neurons, smooth muscle and melanocytes^52,53^. Ectomesenchymal derivatives are underrepresented in these cultures, suggesting that signals from the embryonic environment may be important for their differentiation. One key signalling cue that is important for the emergence of ectomesenchymal identity is pharyngeal endoderm-derived FGF ligands^12^, which recently have been shown to be sufficient for hESC-derived CNCCs to adopt ectomesenchymal identity and upregulate *Twist1*^54^.

Here, we provide mechanistic insight into how intrinsic and extrinsic contributions are integrated to drive ectomesenchymal fate acquisition. We propose that the accumulation of *Twist1* transcripts in cranial neural crest cells is driven by early enhancer activity in combination with potent transcript destabilisation in the ectoderm. As CNCCs migrate ventrally within the developing head, extrinsic signals relieve this transcript destabilisation, enabling rapid activation of the ectomesenchymal GRN programme in the correct spatial context (Fig. 5g). This mechanism reconciles previous observations, including the absence of ectomesenchymal derivatives from CNCCs cultured ex vivo.

An open question is why *Twist1* transcripts are destabilised in the ectoderm but not in other tissues (e.g., extra-embryonic tissue, somites). This could be a result of the differential expression of RNA-Binding Proteins (RBPs) or other regulators, including miRNAs, between tissues of the embryo. Previous work has shown, for example, that there are differences in the transcript-level expression of RBPs between CNCCs at premigratory and migratory stages in development^55^. Alternatively, it is also possible that tissue-specific differences in stability arise through the post-translational modification of RBPs that are more widely expressed. This remains an unexplored mode of transcript regulation in neural crest development.

Consistent with previous studies, the *Twist1* enhancer that we describe here has axially restricted activity, driving transcription in cranial but not trunk neural crest cells. This restriction of enhancer activity may contribute to the intrinsic difference in ectomesenchymal potency of cranial and trunk neural crest cells in amniotes. Together, our findings demonstrate that ectomesenchymal identity emerges through a two-tiered regulatory system: intrinsically through an axially restricted enhancer and *Twist1* transcription, and extrinsically through cues from the ventral head environment that stabilise *Twist1* transcripts to trigger mesenchymal differentiation.

### Evolution of ectomesenchyme as a novel cell type

Ectomesenchyme is a vertebrate innovation; these cells defy germ layer theory, originating in the ectoderm and giving rise to mesenchymal (mesoderm-like) derivatives. In the non-vertebrate chordate *Ciona intestinalis*, it has been proposed that a neural crest-like population of cells exists at the neural plate border. These cells (a9.49 lineage) express some genes that are canonically associated with the NC (*Id, Snail, Ets, Foxd3*) and divide to produce pigment and ependymal cells of the sensory vesicle^56,57^. Nonetheless, the *Ciona* a4.94 lineage lacks ectomesenchymal-like derivatives, suggesting considerable regulatory innovation occurred to give rise to bona fide neural crest with ectomesenchymal derivatives in vertebrate evolution.

In this work, we have shown that across multiple vertebrate lineages, the *Twist1* 3′ UTR sequence is sufficient to potently destabilise transcripts in the neural tube and surface ectoderm. Strikingly, the *twist1a-like* 3′ UTR sequence of the non-vertebrate chordate *Ciona intestinalis* lacks this destabilising activity, underscoring that this regulatory property is a vertebrate-specific acquisition. We have also found that vertebrate *Twist1* 3′ UTRs contain an ARE, UAUUUAUUUAUU. This sequence element closely resembles AREs that have been shown to mediate deadenylation and subsequent degradation of mRNAs in other contexts; it is known to be recognised by RNA-binding proteins (e.g., Tristetraprolin and related proteins) that directly interact with components of the RNA deadenylation machinery^58,59^.

Interestingly, a correlation has been described between 3′ UTR length and morphological complexity (number of cell types) in animal evolution^60^. Consistent with this, we propose that the elaboration of the *Twist1* 3′ UTR sequence, through the introduction of destabilising sequence motifs in vertebrates, was a critical step that facilitated the ability of the ectoderm to give rise to ectomesenchymal derivatives. By preventing precocious or inappropriate differentiation of these cells, this mechanism ensures fidelity of CNCC contributions to the head skeleton. Our findings highlight a novel regulatory innovation that – by restricting the emergence of ectomesenchyme to the correct anatomical context – may have facilitated the ability of neural crest cells to contribute to tissues associated with multiple germ layers.

## Methods

### Animal and embryo husbandry

Fertilised chicken (*Gallus gallus*) eggs were sourced from a local farm (Petaluma Egg Farm, CA) and incubated in a humidified incubator at 37 °C to obtain embryos of the desired stage according to the Hamburger and Hamilton staging chart^61^. Embryos were fixed in 4% PFA in PBS overnight at 4 °C and washed in PBS + 0.1% Tween-20 (PBST).

Wild-type AB strain adult zebrafish (*Danio rerio*) were maintained in a 14/10 h light/dark cycle at 28 °C and fed twice daily. All experiments were approved and conducted in compliance with UC Berkeley Institutional Animal Care and Use Committee (IACUC) protocol AUP-2021-03-14107-1. Embryos were collected and maintained at 28.5 °C in egg water^62^ and staged according to somite number^63^. Following staging, zebrafish embryos were dechorionated with 0.5 mg/mL pronase in egg water for approximately 10–15 min, rinsed 3 times with egg water to remove residual pronase, and fixed in 4% PFA in PBS for 1–2 h at room temperature or overnight at 4 °C.

All mouse husbandry and experiments were carried out in accordance with APLAC institutional guidelines set by the VSC at Stanford University. Wild-type C57Bl/6J mice (Jackson Laboratories) were maintained with standard inbreeding schemes and housed on a 12/12 h light/dark cycle (22 °C, 40–60% RH) with ad libitum access to food and water. For timed pregnancies, male and female mice were housed together beginning at 20:00. Couples were separated, and females were checked for copulatory plugs the following morning between 08:00 and 10:00, with embryonic day 0.5 being established as noon the day of plug. Timed-pregnant dams were sacrificed on the designated day, and embryos were dissected in cold PBS and staged according to Theiler^64^. Following staging, mouse embryos were fixed in 4% PFA in PBS overnight at 4 °C, then washed and stored in PBS at 4 °C until use.

### Electroporation

For *ex ovo* electroporation, embryos were cultured on filter paper according to established protocols^65^. Agar-albumen plates were prepared as follows: 0.6 g of agarose was dissolved in 100 ml of saline solution, cooled to 37 °C, and then combined with 100 ml of thin albumen freshly obtained from eggs, as well as Penicillin-Streptomycin solution (ThermoFisher product 15140122) to 1× concentration. Plasmids were prepared using a Qiagen EndoFree Maxiprep Kit. Plasmid solution (each plasmid at 2 µg/µl in Ringer’s solution with Brilliant Blue dye) was microinjected beneath the epiblast of the HH4 embryo, and a 5.6 V current was applied across the dorsoventral axis of the embryo (5 pulses of 50 ms each). For *in ovo* electroporation, plasmid solution (each plasmid at 2 µg/µl in Ringer’s solution with Brilliant Blue dye) was microinjected into the lumen of the neural tube. A small volume of Ringer’s solution was dropped onto the embryo, and an electrical current was passed from the left to the right of the embryo (21 V current, 3 pulses of 50 ms each). Eggs were sealed with tape and allowed to incubate for an additional 16–20 h.

### Neural crest explants

Mattek glass-bottomed imaging dishes were coated with human fibronectin protein (Biotechne, 1918-FN-02M) as follows: a 25 µg/ml fibronectin solution was made up in PBS using a 1:40 dilution of 1 mg/mL stock solution. 100 µl of fibronectin solution was pipetted onto the glass surface, then the dish was incubated at 37 °C for 1–3 h. Fibronectin solution was removed, and the dish was allowed to fully dry at RT. Dorsal neural folds were micro-dissected in PBS from chicken embryos at HH8-9, then placed onto fibronectin-coated glass dishes containing GMEM (Gibco) + 10% Foetal Bovine Serum (FBS) + 1% Penicillin-Streptomycin solution. Explants were incubated at 37 °C with 5% CO_2_ for 24 h, then fixed in 4% PFA at RT for 20 min.

### Zebrafish transgenesis

Transgene integration was carried out using Tol2-mediated transgenesis. Injection solutions contained 25 ng/μl of the *Twist1*+225kb-E1b: eGFP plasmid and 35 ng/μl of Tol2 mRNA. 2 nl of solution was injected into one-cell-stage AB wild-type embryos. Embryos were then incubated at 23 °C to slow development. The next day, embryos were screened and imaged using a Leica M205FCA fluorescent microscope. Over 40% of injected F0 embryos showed reproducible expression in the neural tube and cranial neural crest cells.

### Enhancer construct cloning

Putative enhancer sequences were ordered as g-blocks with appropriate overhangs (Twist Bioscience) and cloned into the published Sox10 E2:GFP vector^66^. The Sox10 E2 enhancer sequence was removed by successive restriction digestion reactions with KpnI-HF (NEB) and BglII (NEB), then g-blocks and the digested plasmid were assembled by Gibson assembly. Additionally, to test the *Twist1* + 225 kb enhancer in zebrafish, the enhancer sequence was PCR amplified from the above-generated plasmid and cloned into a vector containing Tol2 arms, the E1b minimal promoter, and eGFP using Gibson assembly. For scrambled enhancer construct analyses, the predicted transcription factor binding site within the *Twist1* + 225 kb enhancer sequence was scrambled using a random DNA sequence scrambler (Shuffle DNA tool). To validate that the scrambling of this sequence did not remove or create any additional transcriptional factor binding sites other than the target, the scrambled enhancer sequence was then scanned for TF binding motifs, and the output was compared to the unscrambled enhancer output. All putative and validated enhancer sequences are included in Supplementary Data 3.

### 3′ UTR construct cloning

The destabilised GFP (d2GFP) *β-globin* 3′ UTR construct was a gift from Erica Hutchins^34^. To replace the *β-globin* 3′ UTR with the *Gallus gallus Twist1* 3′ UTR, the *G. gallus Twist1* 3′ UTR sequence (827 bp, Table S3) was ordered as a g-block (Twist Biosciences) with appropriate overhangs to assemble into the plasmid. The *β-globin* 3′ UTR plasmid was digested with NotI and BsaBI, then Gibson assembled with the g-block. The same process was used to clone the *Danio rerio twist1a*, *Mus musculus Twist1*, *Xenopus laevis Twist1.L*, and *Ciona intestinalis twist1a-like* 3′ UTR reporter constructs. To insert the *G. gallus* conserved ARE into the *β-globin* 3′ UTR reporter construct, the plasmid was digested with BglII (NEB), which has a single cut site within the *β-globin* 3′ UTR sequence, and treated with Calf Intestinal Phosphatase (CIP). Complementary oligonucleotides comprising the 30 nt around the ARE were ordered with appropriate overhangs for incorporation at the BglII cut site. Forward and reverse oligos were separately phosphorylated using T4 polynucleotide kinase (PNK) in T4 ligase buffer (37 °C, 1 h), then PNK was heat-inactivated (65 °C, 20 min). Next, oligos were annealed (5 µl each oligo with 90 µl H_2_O) by heating to 95 °C for 3 min and then allowing the reaction mixture to cool slowly to room temperature (1 h). Annealed oligos were ligated into the digested backbone using T4 ligase, then transformed. Colonies were screened for successful insertion using colony PCR.

### Twist1 overexpression construct cloning

To produce the *Twist1* overexpression construct, a codon-optimised g-block encoding the *Gallus gallus* TWIST1 open reading frame (ORF) was obtained from Twist Biosciences. The *β-globin* 3′ UTR plasmid was digested with XhoI and PstI to remove the GFP ORF, followed by Gibson assembly to insert the TWIST1 ORF.

### Hybridisation chain reaction (HCR v3.0)

Chicken embryos were fixed in 4% paraformaldehyde in PBST for two hours at room temperature or overnight at 4 °C, then dehydrated through a methanol: PBST series to 100% methanol and stored at −20 °C. Embryos were rehydrated through a methanol: PBST series and then washed several times in PBST at room temperature. Chicken embryos were digested with proteinase K (NEB, 1 mg/ml in PBST) for 1 min per Hamburger Hamilton stage^61^, quick-washed in PBST, and then postfixed in 4% PFA for 20 min. Embryos were then washed 3 times in PBST for 10 min, twice in 5× SSCT and then pre-hybridised in HCR V3.0 hybridisation buffer for 30 min at 37 °C. Probes were applied at 0.8pmol per 500 µl hybridisation buffer, and embryos were incubated overnight at 37 °C. The next day, embryos were washed 4 times for 15 min each with HCR wash buffer at 37 °C. Embryos were then washed three times with 5× SSCT at room temperature, and then pre-incubated in HCR amplification buffer for 5–30 min at room temperature. HCR hairpins were snap-cooled by heating to 95 °C for 90 s, then cooled to room temperature in darkness. Hairpins were added to the HCR amplification buffer at 4 µl per 500 µl of buffer. Embryos were then incubated in hairpin solution overnight in darkness at room temperature. Finally, embryos were washed 5–8 times (10 min each) in 5× SSCT at room temperature and DAPI-stained. For chicken explants, the above protocol was followed with the following modifications: fixation was for 20 min at RT in 4% PFA, dehydration was in ethanol rather than methanol, and proteinase K treatment and post-fixation were omitted. For zebrafish embryo HCRs, the above protocol was followed with the following modifications: dehydration was in ethanol rather than methanol, and proteinase K treatment and post-fixation were omitted. Probe concentrations were 2 pmol of probe in 500 µl of probe hybridisation buffer. Hairpin concentrations were 6 pmol per hairpin (2 µl each) per 500 µL of amplification buffer. All probes, hairpins, and buffers were purchased through Molecular Instruments.

### Immunohistochemistry

Chicken and mouse embryo sections were immunostained as follows: OCT sections on slides were incubated in Coplin jars in PBS to dissolve OCT (3 × 5 min, RT). Gelatin sections were incubated in Coplin jars in PBS at 37 degrees to dissolve gelatin (1 × 15 min). Slides were then permeabilised in PBS + 0.1% Triton-X (3 × 10 min, RT) before blocking in blocking buffer (PBS + 0.1% Triton-X + 2% donkey serum) (1 h, RT). Primary antibodies were diluted in blocking buffer and applied to slides overnight at 4 °C. Dilutions used were as follows: 1:50 mouse anti-TWIST1 (Abcam ab50887, lot number 1083809), 1:200 goat anti-SOX10 (R&D AF2864, lot number VRY1423081), 1:900 rabbit anti-SOX2 (Abcam ab97959, lot number 1131344-4), and 1:200 goat anti-GFP (Rockland 600-101-215, lot number 51370). The next day, slides were washed 5 times (10 min, RT) in PBS + 0.1% Triton, then incubated in secondary antibody solution for 2 h at RT in darkness. The following secondary antibodies were all used at 1:200 in blocking buffer: donkey anti-mouse Alexa 647 (Invitrogen A31571, lot number 3141854), donkey anti-goat 568 (Invitrogen A11057, lot number 2776028), donkey anti-goat 488 (Invitrogen A11055, lot number 2747580), and donkey anti-rabbit 594 (Invitrogen A21207, lot number 3148833). DAPI was also included at 1:1000. Finally, slides were washed 3 times (10 min, RT) in PBS + 0.1% Triton-X, then coverslipped with Fluoromount (Southern Biotech) and allowed to set overnight at room temperature before confocal imaging.

### Cryosectioning

Chicken embryos (either pre-stained or unstained) were washed in PBS, equilibrated in 5% sucrose in PBS for 30 min, and then in 15% sucrose in PBS overnight at 4 °C. Gelatin solution (7.5% gelatin, 15% sucrose in 0.5× PBS) was melted at 37 °C and applied to embryos, which were then incubated at 37 °C for 3–4 h to allow equilibration. After this incubation, individual embryos were mounted in coffin moulds in gelatin solution, which was allowed to solidify with embryos in the appropriate orientation. After fully solidifying, blocks were flash-frozen in liquid nitrogen, stored at −80 °C, and then cryosectioned at 12 µm on a Leica CM3050 cryostat. Slides were incubated in PBS in a Coplin jar in a 37 °C water bath to dissolve gelatin, then coverslipped with Fluoromount (Southern Biotech) and allowed to set overnight at room temperature before confocal imaging.

E8-8.5 (TS12a) mouse embryos were fixed in 4% PFA (4 °C, overnight) and washed in PBS, then equilibrated in 5% sucrose in PBS for 30 min, and then in 15% sucrose in PBS overnight at 4 °C. Embryos were transferred to room temperature OCT for several minutes to equilibrate, then positioned in OCT in coffin moulds in the appropriate orientation. Blocks were frozen on dry ice, then sectioned at 12 µm on a Leica CM3050 cryostat. Slides were subsequently processed by HCR in situ hybridisation or immunostaining.

### Plastic sectioning of zebrafish embryos

Following whole-mount HCR, zebrafish embryos were washed 3 times for 5 min in PBST and manually deyolked by puncturing the yolk with forceps and gently pipetting up and down to remove yolk granules. Embryos were then embedded in plastic using the JB-4 embedding kit and following a previously published protocol^67^. Embedded embryos were sectioned on a Leitz 1512 microtome at a thickness of 7–10 µm, and sections were adhered to slides by being placed on 20–40 µl droplets of boiled ddH2O on a 50 °C slide warmer. Once the water fully evaporated, slides were mounted in Fluoromount (Invitrogen), coverslipped, and left to set overnight before confocal imaging.

### Confocal imaging

Live embryo images were obtained using a Leica M205 stereoscope. All imaging of fixed tissue was performed using a Zeiss 780 LSM upright confocal microscope. For whole-mount zebrafish HCRs, embryos were mounted in 0.42 μm canyon moulds and imaged with a 20× dipping lens. Image analysis was performed using Fiji^68^, and all images are displayed as summed intensity projections.

### Image quantification

To quantify RFP and GFP signal intensity from widefield images of bilateral scrambled enhancer electroporations, three measurements of corrected total cell fluorescence (CTCF) were taken from the neural tube (NT) and migratory neural crest (NC) regions on either side of the embryo (left and right). These measurements were normalised for each embryo (image) by dividing by the highest value; with this normalisation performed separately for measurements of the neural tube and the migratory neural crest regions, then plotting and unpaired *t*-tests were performed in GraphPad Prism.

To quantify GFP signal intensity from *d2GFP* 3′ UTR bilateral electroporations, sectioned embryos were imaged on a confocal microscope using a 40X oil immersion objective under constant laser intensity and gain settings. Images were processed using FIJI^61,68^ as follows: each Z-stack was converted to a summed-slices intensity projection, then the ‘Threshold Colour’ tool was used to segment RFP-positive pixels (representing successfully electroporated cells on the experimental side of the embryo). The ‘Measure’ tool was used to measure mean pixel intensity in the GFP channel within the segmented RFP-positive region. Next, the image was cropped to only include the control *β-globin* 3′ UTR side of the embryo, then the ‘Threshold Colour’ tool was used to segment and select all GFP-positive pixels (representing successfully electroporated cells). The ‘Measure’ tool was used to measure the mean pixel intensity within the selected region. Finally, the measurements of mean pixel intensities on the left and right halves of the embryo were collated across multiple replicates, and R was used to plot data and perform a paired *t*-test.

To quantify relative GFP fluorescence driven by the d2GFP*-Twist1* 3′ UTR construct in the neural tube and migrating neural crest cells in section images, measurements of CTCF were taken from three small regions of each type in both the RFP and GFP channels per image. Each GFP CTCF measurement was divided by its corresponding RFP CTCF value to account for non-uniformity of transfection efficiency. Measurements were taken in this way for 6 sections across two embryos fixed at HH10-11. R was used to plot the data and perform a Welch *t*-test.

### Computational analysis

#### Hi-ChIP

Raw sequencing reads from previously published H3K27ac Hi-ChIP datasets from HH9 dissected neural folds and whole embryos^21^ were downloaded from the European Nucleotide Archive (accession numbers SRR11719979-SRR11719980). Sequencing reads were mapped to the *galGal6* genome assembly using HiC-Pro^69^, then peaks were called and loop anchoring performed using Hichipper^70^. Output bedpe files from Hichipper were then read into R, and Diffloop^71^ was used to filter contacts (minimum loop size 1.5 kb, minimum of 5 replicates in 3 samples) and perform statistical analysis on neural crest-enriched and depleted loops relative to whole embryo samples. To visualise enhancer-promoter contacts, the Sushi package^72^ was utilised in R.

#### CUT&RUN

Raw sequencing reads from previously published TFAP2A^24^, TFAP2B^24^ and H3K27ac^22^ CUT&RUN experiments were downloaded from the European Nucleotide Archive (accession numbers SRX5402880-SRX5402881; SRX5402884-SRX5402885; SRX7388167-SRX7388168). Raw reads were mapped to the *galGal6* genome assembly using Bowtie2^73^. Replicates for each sample were combined using the samtools merge function^74^, then bigwig files were generated for visualisation purposes using the bamCoverage function from deepTools^75^.

#### ATAC-sequencing

Raw sequencing reads from previously published HH10 and HH12 sorted CNC ATAC-sequencing experiments^23^ were downloaded from the European Nucleotide Archive (accession numbers SRR13295154-SRR13295157). Adaptor sequences (forward: CTGTCTCTTATACACATCT, reverse: AGATGTGTATAAGAGACAG) were trimmed from reads using cutadapt^76^. Trimmed reads were then mapped to the *galGal6* reference genome using Bowtie2^73^. Replicates for each sample were combined using the samtools merge function^74^, then bigwig files were generated for visualisation purposes using the bamCoverage function from deepTools^75^.

#### Visualisation of multimodal sequencing datasets

To visualise ATAC and Cut & Run datasets together over a chromosomal interval of interest, the package PlotGardener in R was utilised^77^.

#### Sequence conservation

The Evolutionarily Conserved Region (ECR) Browser^78^ was used to survey the *Twist1* genomic locus for non-coding sequences that are evolutionarily conserved among vertebrate genomes. Candidate putative enhancer sequences were converted from the *galGal3* genome assembly coordinates to *galGal6* coordinates using the UCSD Genome Browser.

#### Transcription factor binding site analysis

Candidate and/ or validated enhancer sequences were scanned for transcription factor binding motifs using the Find Individual Motif Occurrences (FIMO) tool from MEME Suite^26^. Sequences were analysed against a library of motifs (all redundant vertebrate motifs) downloaded from the JASPAR database^79^.

#### Single-cell RNA-sequencing analysis

Raw read files corresponding to explanted CNCC SMART-seq V4 data^38^ were downloaded from the European Nucleotide Archive (accession numbers SRR6387097–SRR6387192). Each fastq file (corresponding to an individual cell) was mapped to the galGal6 genome assembly using STAR^80^. Individual cell.bam files were then compiled into a single counts table using the featureCounts^81^ programme (via the Subread package). This counts text file was then used to create an anndata object for downstream analysis in Python. Scanpy^82^ was used to normalise, log-transform and scale expression values, then to run a PCA, compute nearest neighbours, cluster cells and embed the neighbourhood graph using UMAP. Expression of key premigratory NC genes, migratory CNCC genes, and ectomesenchymal identity genes was then plotted using the Scanpy pl.heatmap function.

### Reporting summary

Further information on research design is available in the Nature Portfolio Reporting Summary linked to this article.

## Supplementary information

Supplementary Information

Peer Review file

Description of Additional Supplementary Files

Supplementary Data 1

Supplementary Data 2

Supplementary Data 3

Reporting Summary

## Source data

Source Data

## Data Availability

Raw image files are available at the following link: https://figshare.com/s/38f7fd544c8b7899aa03. Source data are provided with this paper.
