# Supplementary material for: Ectomesenchymal identity emerges via relief of Twist1 transcript destabilisation
Source: Nat Commun. 2026 Aug 11;17:9642. doi: 10.1038/s41467-026-76557-6 (PMC13554066; doi:10.1038/s41467-026-76557-6)
Supplement: Supplementary file 1 [file 41467_2026_76557_MOESM1_ESM.pdf]

Supplementary Information

Ectomesenchymal identity emerges via relief of *Twist1* transcript destabilisation

Lara C. Busby, Jessica R. Patrick, Luke W. Lyons, Abby J. Bergman, and Megan L. Martik

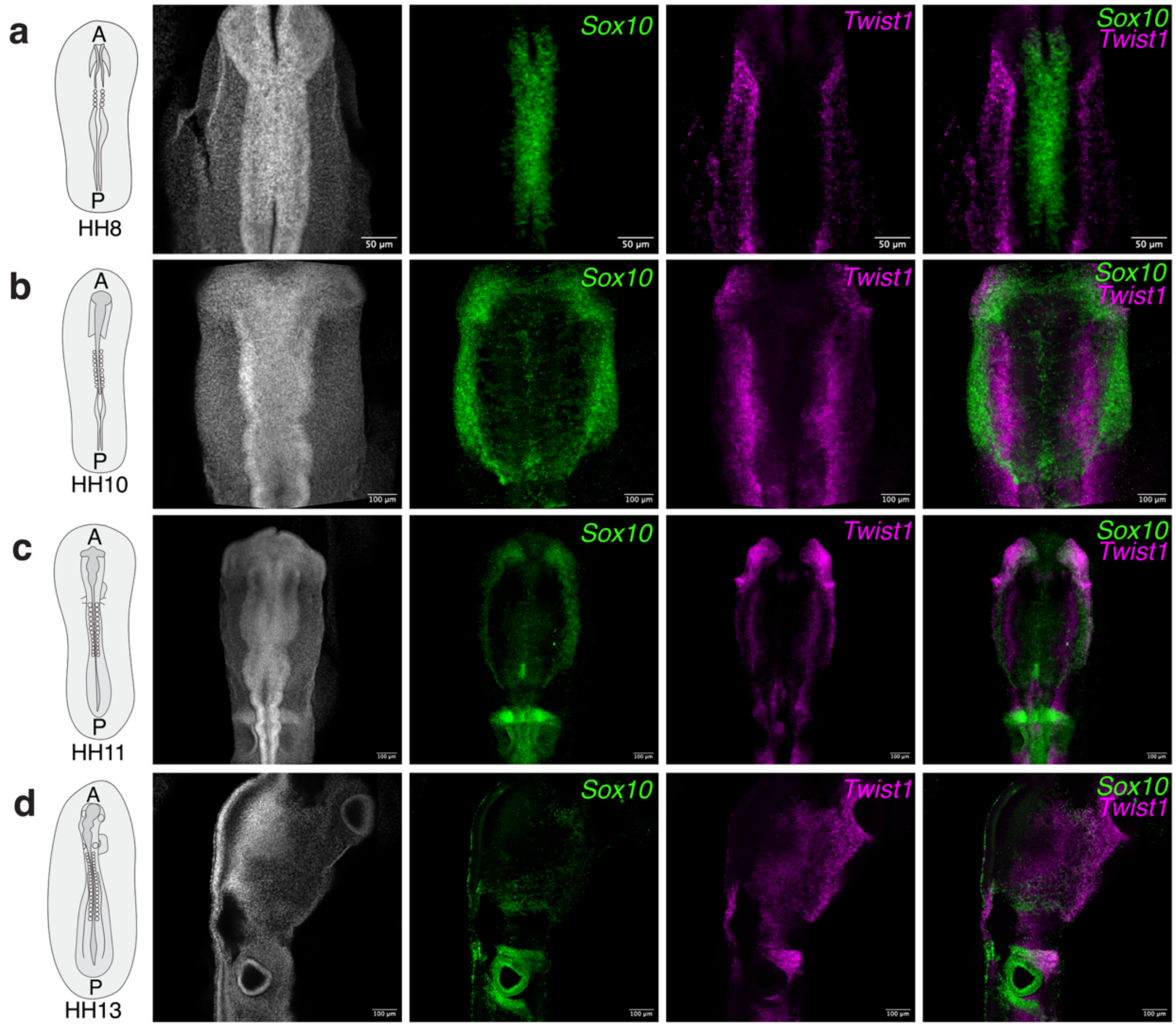

Supplementary Fig.1 | Wholemount HCR staining for *Sox10* and *Twist1* transcripts in chicken. Multiplexed in situ hybridization (V3.0 HCR) for *Sox10* and *Twist1* transcripts in wholemount embryos at **a** HH8 (n=5/5), **b** HH10 (n=5/5), **c** HH11 (n=5/5), and **d** HH12 (n=5/5). All images shown are summed slice intensity projections, with panels showing (from left to right) DAPI nuclear staining, *Sox10* single channel signal, *Twist1* single channel signal and composite *Sox10* and *Twist1*. Scale bars in (**a**) represent 50µm and in (**b**)–(**d**) represent 100µm.

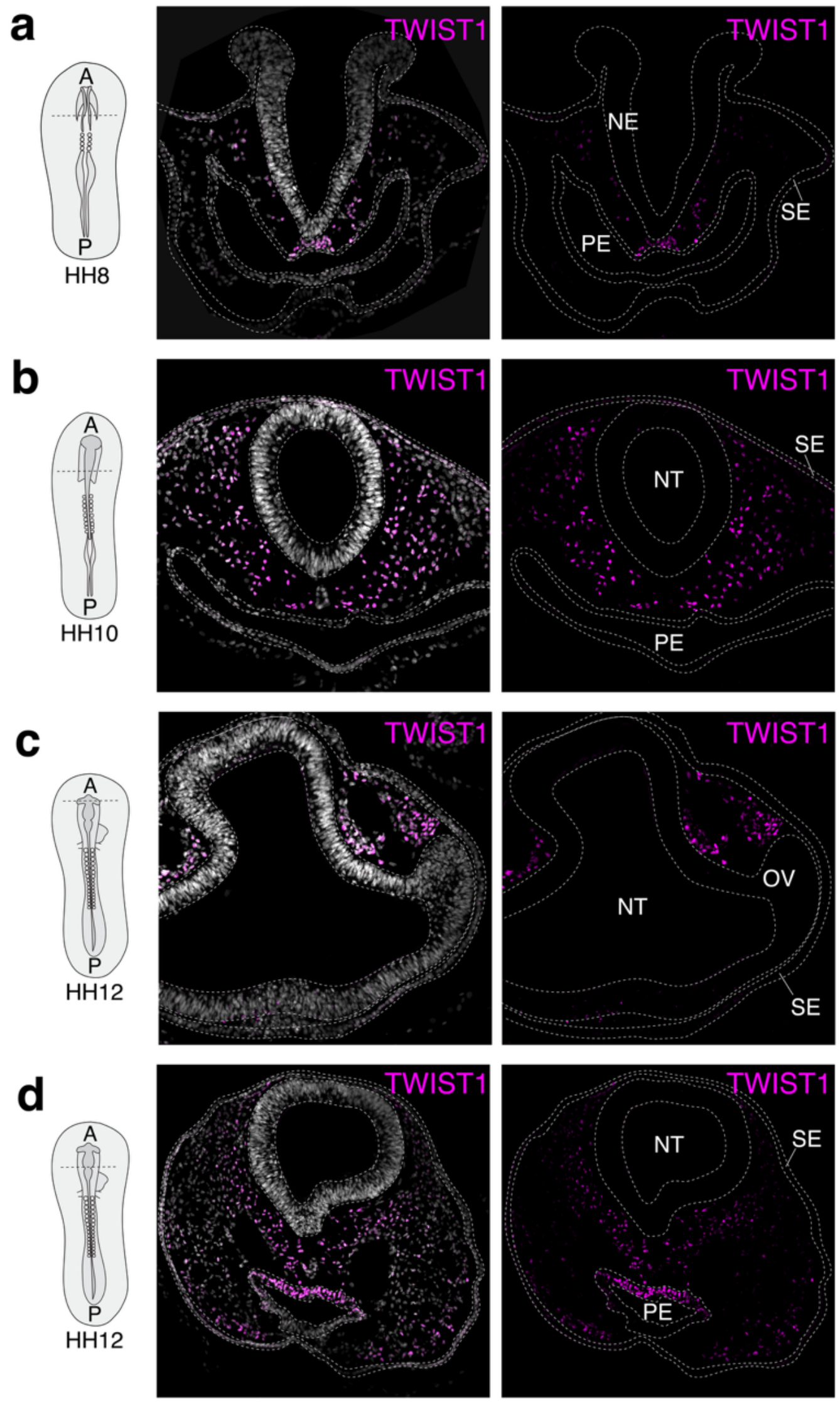

Supplementary Fig.2 | TWIST1 immunostaining in chicken reveals concordance between *Twist1* transcription and protein localisation. Immunostaining for TWIST1 protein in chicken embryo transverse sections at **a** HH8 (n=3/3), **b** HH10 (n=3/3) and **c**–**d** HH12 (n=3/3). In each panel, a schematic representation of the embryo is on the left with a dashed line indicating the axial position of the section. The middle image is a DAPI and TWIST1 composite image and the right image is a TWIST1 single channel image. Acronyms shown are NT: neural tube, SE: surface ectoderm, PE: pharyngeal endoderm, OV: optic vesicle.

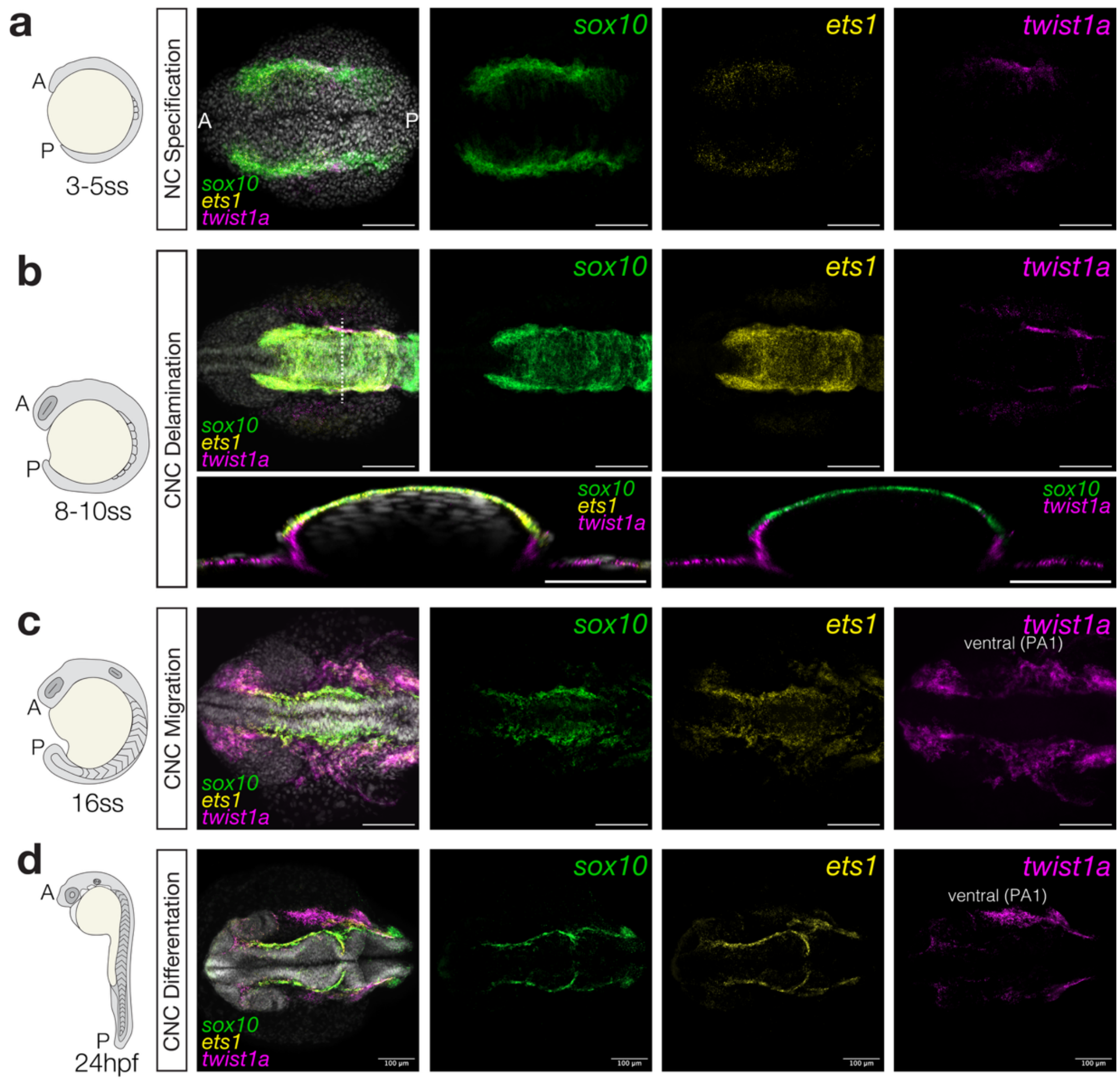

Supplementary Fig.3 | Wholemount HCR staining for *sox10*, *ets1,* and *twist1a* in zebrafish. Multiplexed in situ hybridization (V3.0 HCR) for *sox10*, *ets1*, and *twist1a* in wholemount zebrafish embryos at **a** 3-5ss (n=10/10) **b** 8-10ss (n=9/9), **c** 16ss (n=8/8), and **d** 24hpf (n=3/3). All images shown are summed slice intensity projections, with panels showing (from left to right) a composite image (DAPI, *sox10*, *ets1,* and *twist1a*), followed by single channel images of *sox10, ets1,* and *twist1a*. **b** also displays resliced images along the yz-plane at the axial level indicated by the white dotted line in the upper left panel, showing a composite image followed by a double-channel image of *sox10* and *twist1a*. Acronyms: A, anterior; P, posterior; PA1, pharyngeal arch 1. Scale bars for resliced images are 50µm and all other scale bars represent 100µm.

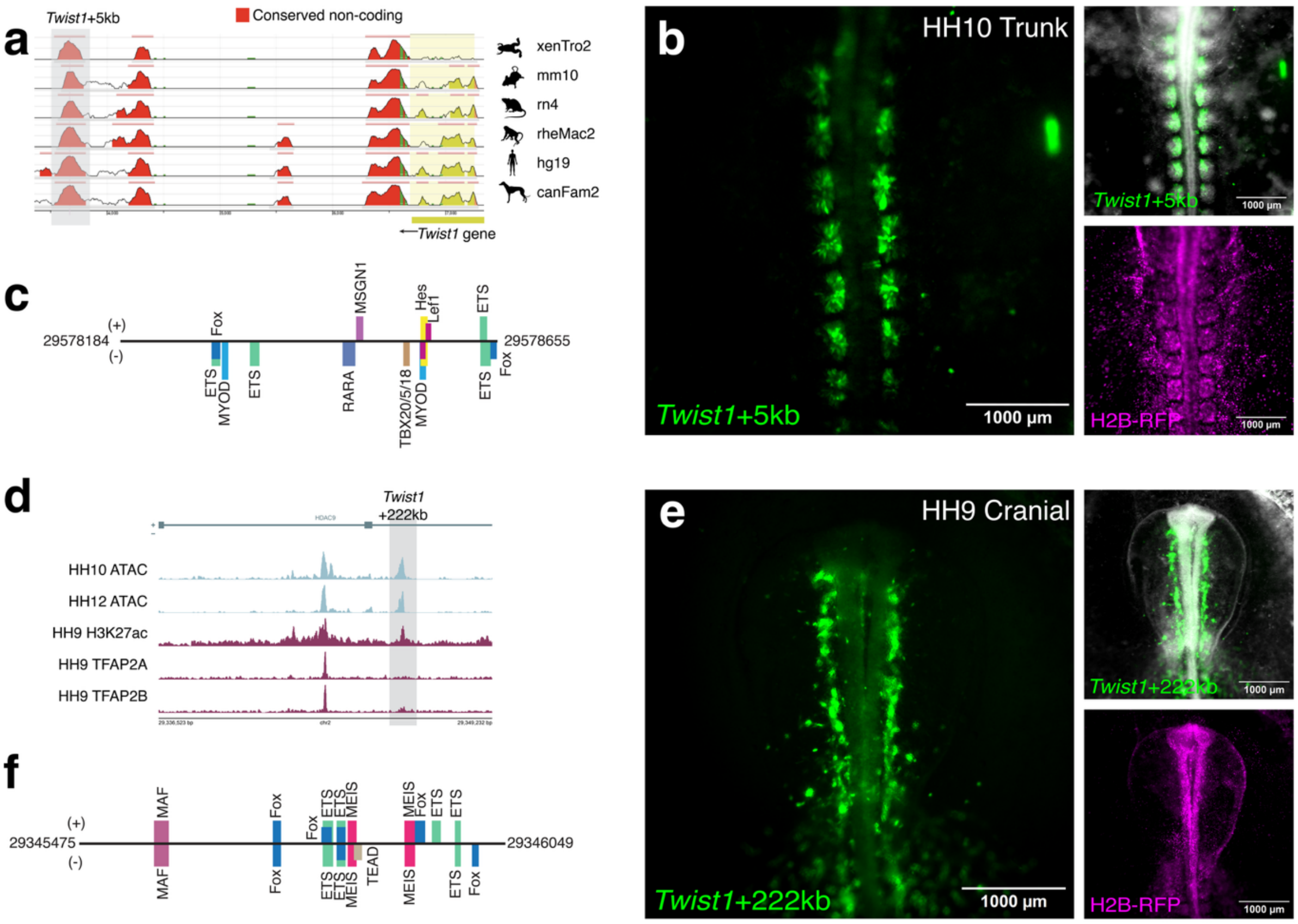

Supplementary Fig.4 | Validated mesodermal enhancers for *Twist1*. a Conserved sequence plot from the ECR Browser in the genomic region just downstream of the *Twist1* gene (base genome galGal3), with red peaks indicating regions of sequence homology between vertebrate genomes and galGal3 genome sequence. The *Twist1*+5kb enhancer peak is indicated by the grey shaded box. **b** Fluorescence images showing (left) GFP fluorescence, (upper right) composite brightfield and GFP fluorescence and (lower right) H2B-RFP fluorescence in a representative embryo at HH10. GFP fluorescence is detected in the medial portion of the somites (n=6/6). **c** Box diagram of the *Twist1*+5kb enhancer with predicted transcription factor binding sites indicated. **d** Genome tracks showing chromatin accessibility, H3K27ac and TFAP2 transcription factor binding in the region encompassing the *Twist1*+222kb enhancer. Note that this enhancer is contained within the same Hi-ChIP contact with the *Twist1* promoter region as *Twist1*+225kb (see main text Fig. 2). **e** Fluorescence images showing (left) GFP fluorescence, (upper right) composite brightfield and GFP fluorescence image and (lower right) H2B-RFP fluorescence in a representative embryo at HH9. GFP fluorescence is detected in cranial mesoderm close to the midline (n=9/9). **f** Box diagram of the *Twist1*+222kb enhancer with predicted transcription factor binding sites indicated.

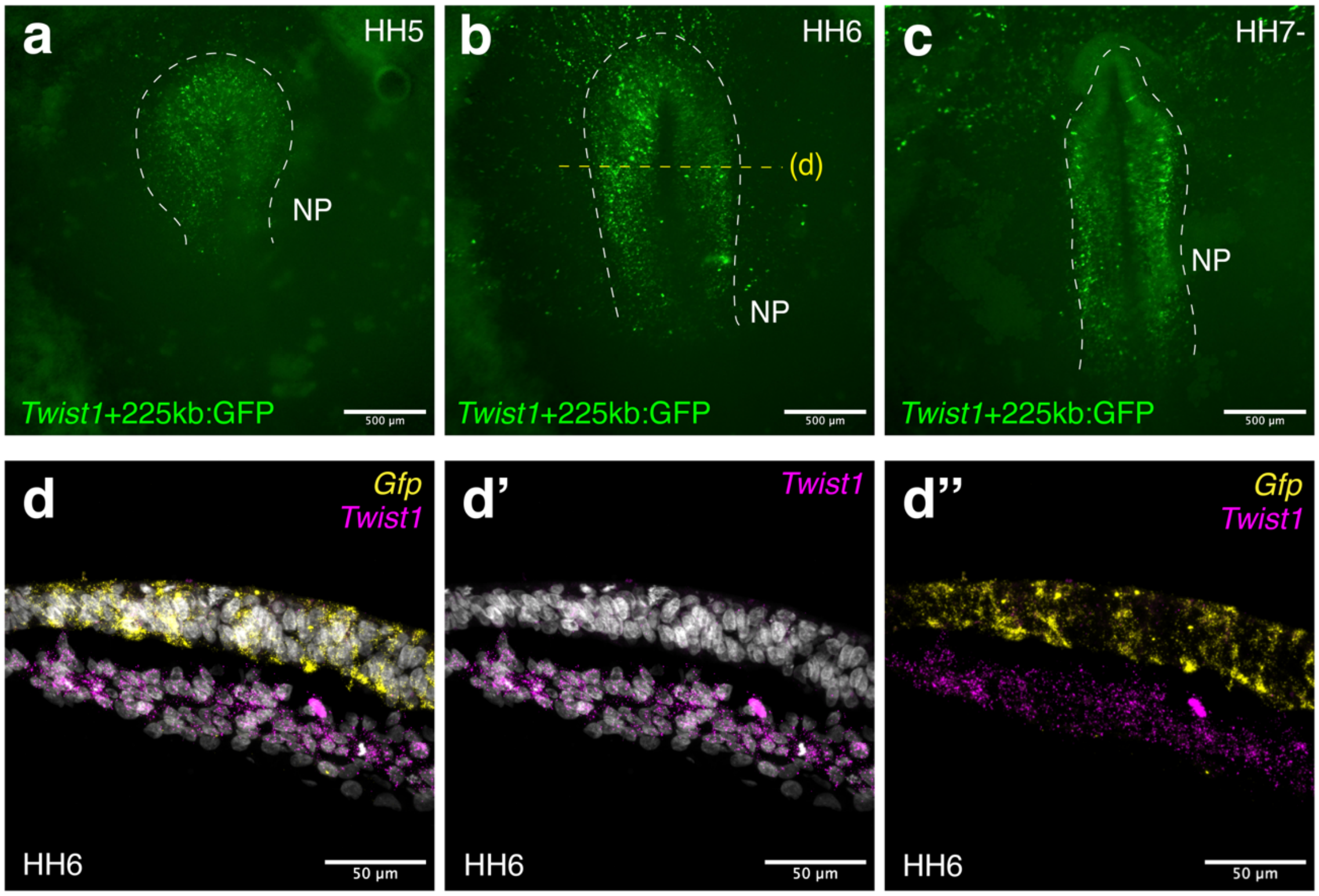

Supplementary Fig. 5 | Early *Twist1*+225kb enhancer activity. a-**c** Representative images of live embryos showing the activity of *Twist1*+225kb: GFP in the neural plate as early as 6 hours after electroporation. Images are at HH5 (**a**, n=6/6), HH6 (**b**, n=4/4) and HH7- (**c**, n=2/2). The approximate location of the neural plate is indicated by the dashed white lines. The yellow dashed line in **b** indicates the position of the section shown in **d**. **d** HCR-stained enhancer embryo at HH6 – **d** is a composite of *Gfp* and *Twist1* transcripts with DAPI, **d'** shows *Twist1* transcripts overlaid with DAPI and **d''** is a composite of *Twist1* and *Gfp* transcripts. *Gfp* transcripts are detected in the neural plate (dorsal cell layer, top of image) while *Twist1* transcripts are primarily detected in the ventral mesodermal cell layer. Image is representative of the 9 sections imaged across 3 embryos.

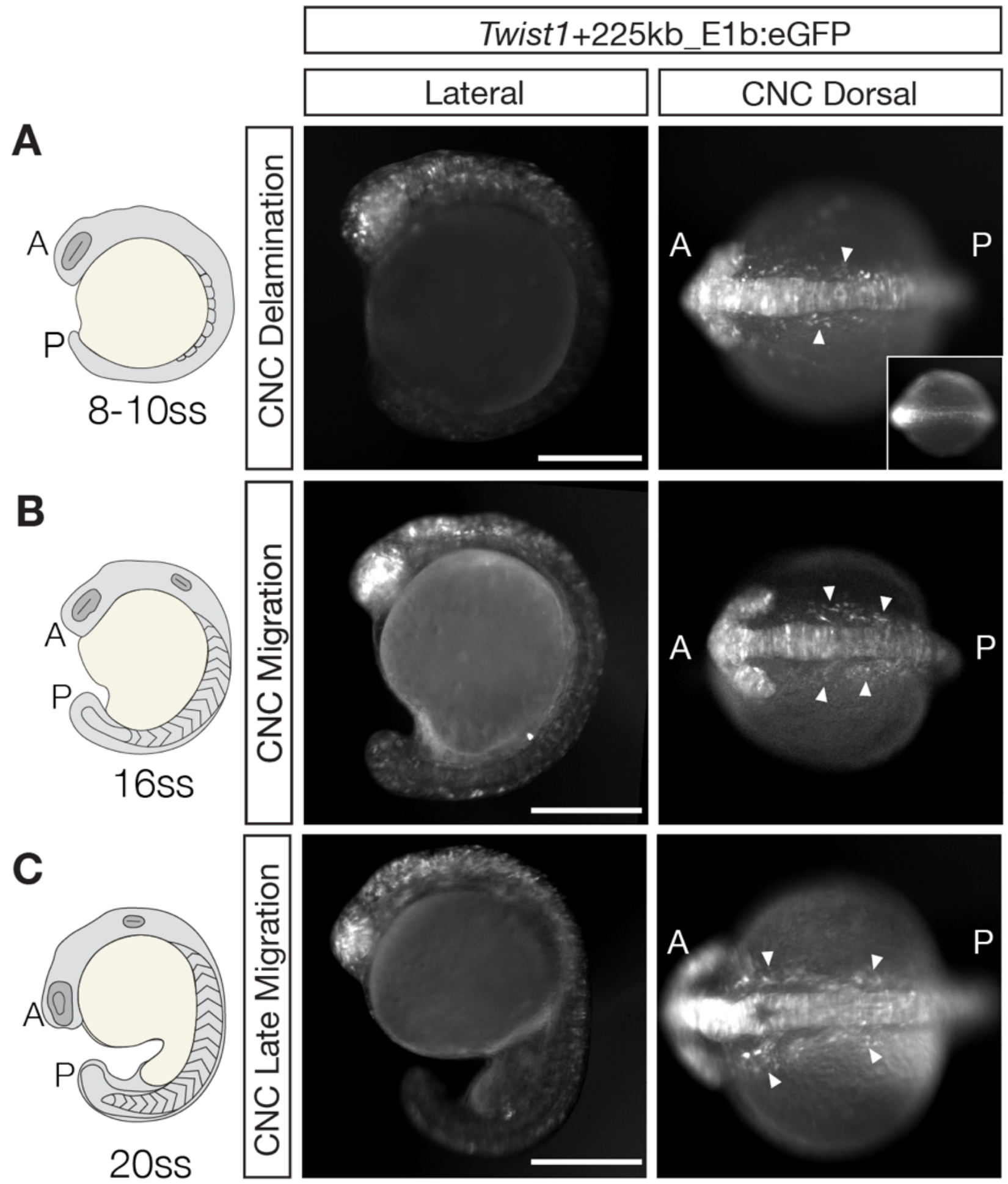

Supplementary Fig.6 | *Twist1*+225kb drives comparable expression in zebrafish.
Representative fluorescent images of F0 injected *GgTwist1*+225kb-E1b:eGFP zebrafish embryos (n=~40/100) across neural crest development at **a** 8-10ss, **b** 16ss, and **c** 20ss. Embryos are positioned both laterally to show expression across the entire body axis as well as dorsally to show the cranial neural crest axial region. The inset in panel **a** is a dorsal view of the same embryo in the trunk region, showing minimal to no GFP expression. White arrowheads indicate migrating cranial neural crest cells. Abbreviations: A, anterior; P, posterior. Scale bars represent 1mm.

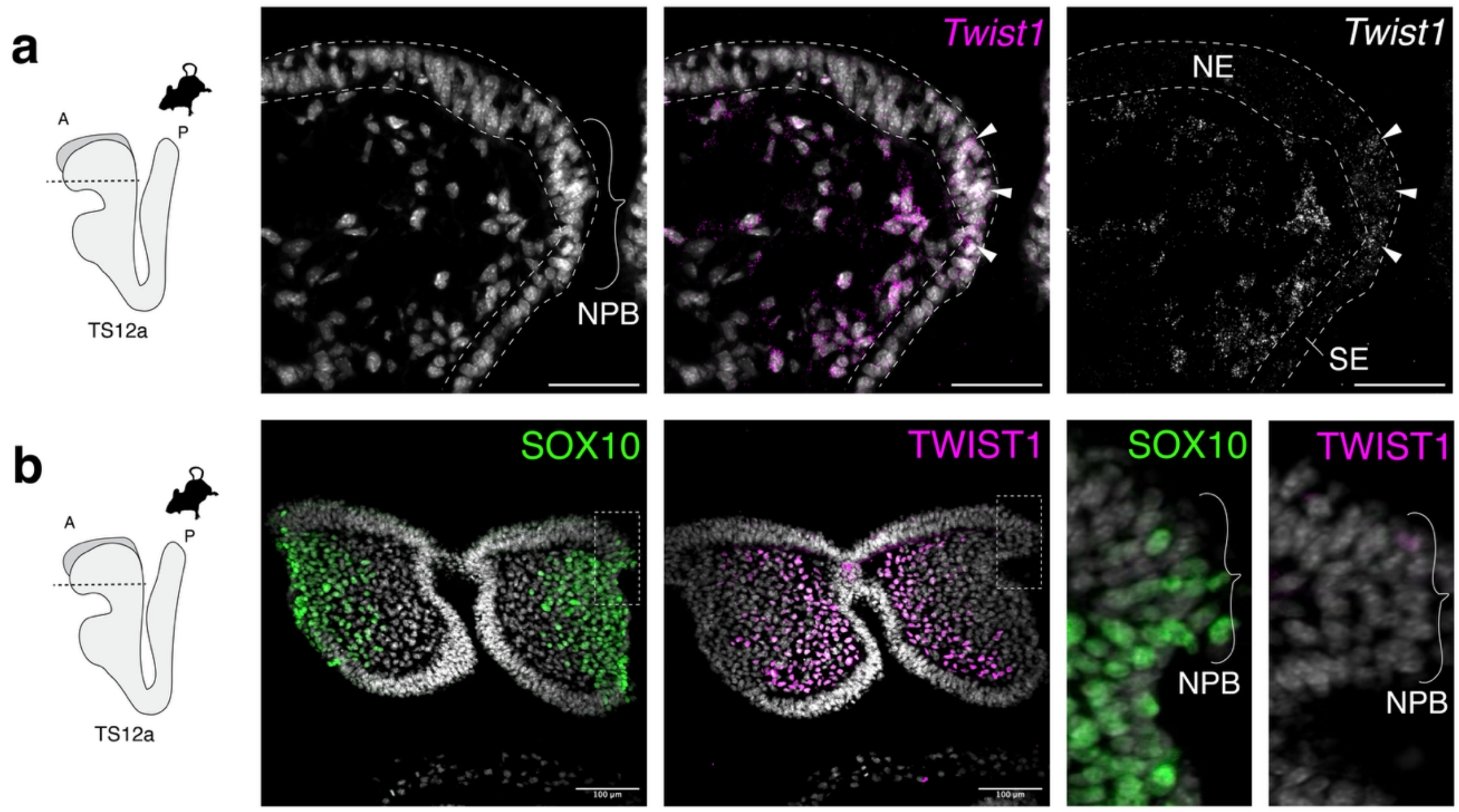

Supplementary Fig.7 | TWIST1 protein is absent from premigratory and early migratory CNCCs in mouse despite detectable *Twist1* transcript. a HCR *in situ* hybridization for *Twist1* in transverse sections of mouse embryo at TS12a (E8.25) (n=3/3). The left panel is a schematic of the TS12a mouse embryo with the position of transverse section shown by the dashed line. Remaining panels from left to right show: DAPI single channel image, *Twist1* HCR stain and DAPI composite image and *Twist1* HCR stain single channel image. The position of the neural plate border (NPB) is indicated by the bracket. Arrowheads indicate cells in the NPB with *Twist1* transcript expression. **b** Immunostaining for SOX10 and TWIST1 protein in transverse serial sections of a mouse embryo at TS12a (E8.25) (n=3/3). The left panel is a schematic of the TS12a mouse embryo with the position of transverse sections shown by the dashed line. The remaining panels from left to right show: DAPI and SOX10 composite image, DAPI and TWIST1 composite image, then inset enlarged images of the SOX10 and DAPI composite image and the TWIST1 and DAPI composite image. Dashed white boxes indicate the position of inset enlarged images, and the bracket indicates the position of the neural plate border (NPB). Scale bars in (**a**) represent 50μm and in (**b**) represent 100μm.

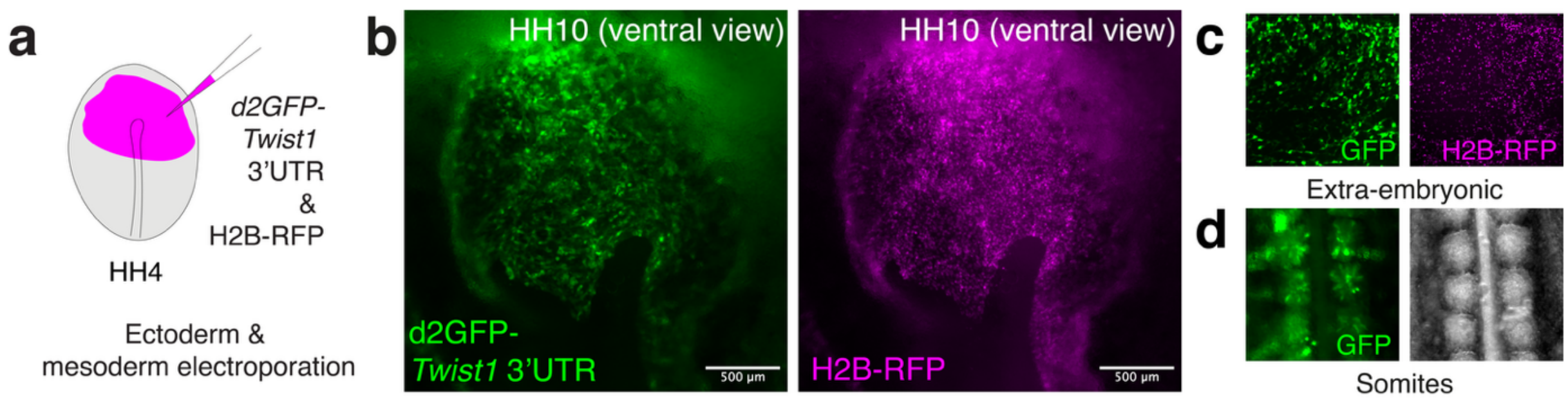

Supplementary Fig. 8 | The post-transcriptional destabilisation of *Twist1* transcripts is tissue-specific. Schematic representation of whole ectoderm electroporation. **b** Single channel images of a representative HH10 embryo in ventral view with GFP signal shown on the left and RFP signal shown on the right. **c** Single channel images of extra-embryonic tissue showing GFP signal (left) and RFP signal (right). **d** Single channel images of trunk region showing GFP signal (left) in somite tissue – right image shows a brightfield overview.
