# Supplementary material for: Ectomesenchymal identity emerges via relief of Twist1 transcript destabilisation
Source: Nat Commun. 2026 Aug 11;17:9642. doi: 10.1038/s41467-026-76557-6 (PMC13554066; doi:10.1038/s41467-026-76557-6)
Supplement: Supplementary file 2 [file 41467_2026_76557_MOESM2_ESM.pdf]

nature portfolio

Peer Review File

Ectomesenchymal identity emerges via relief of *Twist1* transcript destabilisation

Corresponding Author: Professor Megan Martik

This file contains all reviewer reports in order by version, followed by all author rebuttals in order by version.

Version 0:

Reviewer comments:

Reviewer #1

(Remarks to the Author)
Busby et al. identify a distal enhancer that activates twist1 expression in the neural tube and dorsal ectoderm, as well as a 3' UTR element that destabilizes this expression prior to migration of cranial neural crest cells into ventral head regions where they form ectomesenchyme.

Previous studies identified twist1 expression in cranial neural crest, even though it is primarily expressed in mesodermal and mesenchyme derivatives. The identification of a remote twist1 enhancer located ~225 kb upstream of the gene is a significant finding. Interestingly, the authors show that this enhancer is not sufficient for robust expression due to one or more RNA destabilization elements located in the twist1 3' UTR. Relief of this destabilization may be mediated by FGF signals after migration of cranial neural crest cells to appropriate target sites.

The authors make a compelling case that the ectomesenchyme is a novel vertebrate cell type not seen in invertebrates, including invertebrate chordates such as Ciona. It was once believed that the entire neural crest was a novel cell type in vertebrates, although it is now generally accepted that a neural crest lineage is present in Ciona (Abitua et al. 2012; Todorov et al. 2024). However, ectomesenchyme is clearly absent in Ciona, although an ectomesenchyme-like cell type was obtained in Ciona tadpoles and juveniles upon misexpression of Twist in the neural crest lineage using a Mitf enhancer.

As the authors note, the role of Twist1 in cell migration and EMT is a bit uncertain since it is primarily a mesenchyme determinant. A key feature of the mesenchyme developmental program involves upregulation of Snail (and related genes), which in turn mediates EMT. The authors should revise the Discussion to include some of the points raised above and emphasize the mechanisms they identified in this study: evolution of a key neural crest cell type, ectomesenchyme, through the activation of a mesenchyme determinant in ectodermal derivates by a remote twist1 enhancer and the modulation of this activity by post-transcriptional attenuator elements in the 3' UTR.

Reviewer #2

(Remarks to the Author)
This manuscript from Busby et al., examines the regulation of the Twist1 transcription factor gene in cranial neural crest (CNCC) development. By reanalysis of existing H3K27ac HI-ChIP, ATAC-seq and Cut & Run for CNCC transcriptional regulators the authors identify and then experimentally confirm a CNCC-specific enhancer for Twist1 in the chick and zebrafish which is regulated by NCC core transcription factors. While this enhancer drives early reporter expression, Twist1 mRNA is not detected until later stages in post-migratory NCCs leading to the central assertion that contrary to current assumptions, Twist1 may not be a regulator of early NCC EMT but rather is important only in post-migratory NCC differentiation. The authors identify specific 3'UTR sequences that are conserved across vertebrates and contribute to Twist1 instability. This manuscript makes the important and convincing observation that Twist1 mRNA expression is observed later than presumed revealing a gap in our understanding of its function in NCC development. That Twist1 mRNA stability may be specifically regulated in the neural crest is interesting and potentially important. Overall, the data appear to be of high quality, and the manuscript is very clearly written. Unfortunately, as currently presented, this study falls short of demonstrating that observed Twist1 mRNA expression changes during NCC development are attributable to mRNA stability rather than simple differences between the activity of this one enhancer and the Twist1 gene. Equally importantly, it remains

unknown whether there is a developmental function of such post-transcriptional regulation if it occurs. This could be examined by overexpression of (stabilized) Twist1 in early NCCs, or more ideally by performing experiments to disrupt the ARE sequence to examine the consequences on NCC development (though this would be technically challenging). In addition to these core issues, several more minor points might also be considered to strengthen the manuscript.

• Further support of the core hypothesis that Twist1 mRNA stability is transiently decreased by the identified ARE element would be provided by evidence that transcripts with just the Twist1 3'UTR are also upregulated in post-migratory NCCs. For example, does adding the Twist1 3'UTR to the Twist1+225kb: GFP reporter result in early destabilization with upregulation in postmigratory NCCs?

• That cultured CNC explants do not appear to upregulate Twist1 mRNA is not strong evidence of an external cue that specifically regulates Twist1 stability. This difference could be a consequence of cells failing to fully/properly differentiate and/or begin to express NCC-derivative gene programs. Evidence that explants are otherwise transcriptionally similar to in vivo NCCs would be needed to make conclusions that signaling environment regulates Twist1 mRNA stability.

• In experiments presented in Figure 4 the Twist1 3'-UTR is compared with the B-globin 3'UTR for its ability to stabilize transcript. However, it is known that B-globin mRNA has extraordinary stability conferred through the 3'UTR, so an additional control comparison may be appropriate. This concern is alleviated somewhat by the observations that non-NCCs do not exhibit transcript destabilization and that the Twist1 ARE can destabilize B-globin but then begs the question- why does Twist1 ARE not destabilize Twist1 outside of NCCs? Identifying NCC-specific regulators that bind Twist1 ARE is probably beyond the scope of this manuscript, but some discussion should be given to this.

• The investigators examine mouse embryos and report that Twist1 mRNA but not protein is found in premigratory and early migratory CNCCs, but if I understand correctly, this is a different situation than chick which only has detectable Twist1 transcript in late migratory CNCCs. Given conservation of the Twist1 ARE, this is somewhat surprising. This significance of this apparent difference (possibly relating to differences in NCC timing in mouse and chick?) might be discussed.

Minor points

• I could not open Supplemental Data tables 1-3 which indicated an invalid file format.

• For in vivo enhancer experiments presented in Fig. 2c-h' I could not locate number of embryos that were scored. I was particularly curious for this information relating to the trunk neural tube vs. NCCs.

• In addition to TFAP2 and FOXD3, several other predicted regulators of the Twist1+225kb enhancer were predicted by FIMO, including HMGA1 were these tested by generation of modified enhancer constructs and found not to have an effect, or were these candidate regulators untested?

Version 1:

Reviewer comments:

Reviewer #2

(Remarks to the Author)

The modifications have greatly improved the manuscript. In particular, functional experiments expressing a stabilized Twist1 transcript reveal the importance of its proper (de)stabilization. This manuscript provides important insights into Twist1 regulation and clarifies functions in the development of the neural crest lineage.

Open Access This Peer Review File is licensed under a Creative Commons Attribution 4.0 International License, which permits use, sharing, adaptation, distribution and reproduction in any medium or format, as long as you give appropriate credit to the original author(s) and the source, provide a link to the Creative Commons license, and indicate if changes were made.
In cases where reviewers are anonymous, credit should be given to 'Anonymous Referee' and the source.
The images or other third party material in this Peer Review File are included in the article's Creative Commons license, unless indicated otherwise in a credit line to the material. If material is not included in the article's Creative Commons license and your intended use is not permitted by statutory regulation or exceeds the permitted use, you will need to obtain permission directly from the copyright holder.
To view a copy of this license, visit https://creativecommons.org/licenses/by/4.0/

Response to Reviewers

We thank both reviewers for their thoughtful reading and evaluation of our manuscript. We have incorporated their suggestions to the benefit of the overall story and strength of the manuscript.

For clarity, please find a list of the figure changes we have made below:

- Added additional panels (f) and (g) to Figure 4, showing the presence of nascent *Twist1* transcripts throughout the neural tube at HH8-9. The boxplot in Fig. 4e was replaced for stylistic consistency (the underlying data is unchanged).
- Added an additional Supplementary Figure – SF5 – which shows the activity of the *Twist1*+225kb: GFP enhancer reporter at HH5-7 throughout the neural plate.
- Added an additional main text figure – New Figure 5 – which examines the impact of TWIST1 overexpression in the neural plate.
- Swapped out wholemount images in the original Figure 5 (now Figure 6) for sectioned UTR reporter images and added additional panels showing our re-analysis of explant single cell RNA-sequencing data.
- Moved the wholemount images in original Figure 5 to a new Supplementary Figure, SF8.

All modification to the text is indicated by tracked changes in the revised manuscript and is referenced by **line numbers** in the response below.

Reviewer #1 (Remarks to the Author):

Busby et al. identify a distal enhancer that activates twist1 expression in the neural tube and dorsal ectoderm, as well as a 3' UTR element that destabilizes this expression prior to migration of cranial neural crest cells into ventral head regions where they form ectomesenchyme.

Previous studies identified twist1 expression in cranial neural crest, even though it is primarily expressed in mesodermal and mesenchyme derivatives. The identification of a remote twist1 enhancer located ~225 kb upstream of the gene is a significant finding. Interestingly, the authors show that this enhancer is not sufficient for robust expression due to one or more RNA destabilization elements located in the twist1 3' UTR. Relief of this destabilization may be mediated by FGF signals after migration of cranial neural crest cells to appropriate target sites.

The authors make a compelling case that the ectomesenchyme is a novel vertebrate cell type not seen in invertebrates, including invertebrate chordates such as Ciona. It was once believed that the entire neural crest was a novel cell type in vertebrates, although it is now generally accepted that a neural crest lineage is present in Ciona (Abitua et al. 2012; Todorov et al. 2024). However, ectomesenchyme is clearly absent in Ciona, although an ectomesenchyme-like cell type was obtained in Ciona tadpoles and juveniles upon misexpression of Twist in the neural crest lineage using a Mitf enhancer.

As the authors note, the role of Twist1 in cell migration and EMT is a bit uncertain since it is primarily a mesenchyme determinant. A key feature of the mesenchyme developmental program involves upregulation of Snail (and related genes), which in turn mediates EMT. The authors should revise the Discussion to include some of the points raised above and emphasize the mechanisms they identified in this study: evolution of a key neural crest cell type, ectomesenchyme, through the activation of a mesenchyme determinant in ectodermal derivates by a remote twist1 enhancer and the modulation of this activity by post-transcriptional attenuator elements in the 3' UTR.

We thank Reviewer 1 for their thoughtful comments contextualizing our results in vertebrate evolution. To address these comments, we have added some additional text to the discussion section highlighting the potential implications of our results for the evolutionary history of neural crest. As part of this, we cite key studies that have examined neural crest-like cells in *Ciona intestinalis,* namely Abitua et al., 2012 & Todorov et al., 2024, as highlighted by the reviewer. This text can be found at lines **416-422** of the revised manuscript.

Reviewer #2 (Remarks to the Author):

This manuscript from Busby et al., examines the regulation of the Twist1 transcription factor gene in cranial neural crest (CNCC) development. By reanalysis of existing H3K27ac HI-ChIP, ATAC-seq and Cut & Run for CNCC transcriptional regulators the authors identify and then experimentally confirm a CNCC-specific enhancer for Twist1 in the chick and zebrafish which is regulated by NCC core transcription factors. While this enhancer drives early reporter expression, Twist1 mRNA is not detected until later stages in post-migratory NCCs leading to the central assertion that contrary to current assumptions, Twist1 may not be a regulator of early NCC EMT but rather is important only in post-migratory NCC differentiation. The authors identify specific 3'UTR sequences that are conserved across vertebrates and contribute to Twist1 instability. This manuscript makes the important and convincing observation that Twist1 mRNA expression is observed later than presumed revealing a gap in our understanding of its function in NCC development. That Twist1 mRNA stability may be specifically regulated in the neural crest is interesting and potentially important. Overall, the data appear to be of high quality, and the manuscript is very clearly written.

Thank you for this accurate summary of our work.

Unfortunately, as currently presented, this study falls short of demonstrating that observed Twist1 mRNA expression changes during NCC development are attributable to mRNA stability rather than simple differences between the activity of this one enhancer and the Twist1 gene.
Equally importantly, it remains unknown whether there is a developmental function of such post-transcriptional regulation if it occurs. This could be examined by overexpression of (stabilized) Twist1 in early NCCs, or more ideally by performing experiments to disrupt the ARE sequence to examine the consequences on NCC development (though this would be technically challenging). In addition to these core issues, several more minor points might also be considered to strengthen the manuscript.

Thank you for your suggestions. We have addressed this comment by performing overexpression of the *Twist1* ORF followed by the *β-globin* 3' UTR. Additional context is given to this experiment by our observation of nuclear *Twist1* transcripts throughout the neural tube (discussed in detail below).

As the *Twist1*+225kb enhancer is active throughout the neural tube (Fig. 2c-f), and nuclear *Twist1* transcripts are detectable (Fig. 4f-g), we decided to investigate the consequence of overexpressing stabilised *Twist1* transcripts throughout the prospective neural plate from HH4-5 (Fig. 5a-b). Electroporation of our overexpression construct results in the accumulation of TWIST1 protein within 6 hours (new Fig. 5c). When we raised these embryos to HH8-12, we found that many embryos exhibited a reproducible neural tube closure defect with an open neural plate in

the cranial region but normal neural tube morphology in the trunk region (new Fig. 5d). This result suggested that expression of stabilised *Twist1* transcripts in the neural plate is sufficient to disrupt its normal morphogenesis. We stained sections of these embryos for SOX2 and TWIST1 protein, finding that many nuclei enriched with TWIST1 protein had little SOX2 protein relative to their neighbours in the neuroepithelium (new Fig. 5e-f).

Despite the disruption to neuroepithelial morphogenesis in these embryos, we did observe cells lateral to the neural plate ingressing from the epithelium (arrowhead in Fig. 5e) (n=3/3 embryos), suggesting that the neural crest is still able to form upon precocious TWIST1 activity. Thus, the importance of *Twist1* transcript destabilisation appears two-fold: 1) to protect neural progenitors from loss of identity and neural tube malformation, and 2) to restrict TWIST1 activity to the subset of cranial neural crest cells that are in the appropriate position to form ectomesenchyme. The text discussing these experiments can be found in the revised manuscript at lines **221-237**.

• Further support of the core hypothesis that Twist1 mRNA stability is transiently decreased by the identified ARE element would be provided by evidence that transcripts with just the Twist1 3'UTR are also upregulated in post-migratory NCCs. For example, does adding the Twist1 3'UTR to the Twist1+225kb: GFP reporter result in early destabilization with upregulation in postmigratory NCCs?

Thank you for this suggestion. We repeated our injections of the *Twist1* 3' UTR reporter construct, sectioned the embryos, then amplified the GFP signal by antibody staining. Here, despite labelling of cells in the neural tube with the H2B-RFP plasmid, GFP is primarily detected in the surface ectoderm and migrating cranial neural crest cells (Fig. 6a-c). We quantified GFP fluorescence intensity relative to the co-electroporated H2B-RFP fluorescence intensity, finding that relative GFP fluorescence is significantly higher in migrating CNCCs than in cells in the neural tube (Fig. 6c). These data suggest that the *Twist1* 3' UTR is more stable in in late migratory CNCCs than in the neural tube. These data are discussed in the revised text at lines **256-263**.

• That cultured CNC explants do not appear to upregulate Twist1 mRNA is not strong evidence of an external cue that specifically regulates Twist1 stability. This difference could be a consequence of cells failing to fully/properly differentiate and/or begin to express NCC-derivative gene programs. Evidence that explants are otherwise transcriptionally similar to in vivo NCCs would be needed to make conclusions that signaling environment regulates Twist1 mRNA stability.

Thank you for this comment. The explanting of dorsal neural folds on fibronectin has been widely used to study neural crest cells, where they are known to be successfully specified, undergo EMT and migrate away from the neuroepithelial tissue (Bronner-Fraser and Garcia-Castro, 2008). In this study, we have shown that CNCCs in culture express canonical NC specifier genes (*Sox10, Ets1*) and adopt a mesenchymal morphology, suggesting that they are *bona fide* neural crest cells.

To support this, we have also analysed a previously published single cell RNA-sequencing dataset of explanted CNCCs at 24 hours (Morrison et al., 2017). We include this data in the revised paper (new Figure 6, text in lines **271-278**) showing that explanted CNCCs in culture express numerous canonical markers identified in previous studies (Simoes-Costa et al., 2014),

including *Snai2, Sox10, Foxd3, Ets1, Rxrg, Col9a3, Nrp2, Ltk, Dact2, Pdgfra, Lmo4, Tfap2a, Tfap2b* and *Cdh11* (Fig. 6f). Importantly, this transcriptomic data corroborates the absence of ectomesenchymal marker expression in explanted CNCCs, including *Twist1, Prrx1/2, Fli1,* and *Dlx* genes (Fig. 6f).

Together, our observations suggest that explanted CNCCs at 24 hours of culture are *bona fide* neural crest cells maintained in an early migratory gene expression state that do not undergo a switch to ectomesenchymal identity. This is consistent with the known requirement for extrinsic signalling for ectomesenchymal identity acquisition (Blentic et al., 2008) as well as other explant experiments that have shown very few ectomesenchymal derivatives differentiate in these cultures (Bhattacharya et al., 2018; Cohen and Konisberg, 1975). Taken with our other observations, we suggest that this indicates an inability for CNCCs to undergo the transition from multipotent progenitor to ectomesenchymal progenitor and implicates a requirement for extrinsic signals for *Twist1* mRNA accumulation to occur.

• In experiments presented in Figure 4 the Twist1 3'-UTR is compared with the B-globin 3'UTR for its ability to stabilize transcript. However, it is known that B-globin mRNA has extraordinary stability conferred through the 3'UTR, so an additional control comparison may be appropriate. This concern is alleviated somewhat by the observations that non-NCCs do not exhibit transcript destabilization and that the Twist1 ARE can destabilize B-globin but then begs the question- why does Twist1 ARE not destabilize Twist1 outside of NCCs? Identifying NCC-specific regulators that bind Twist1 ARE is probably beyond the scope of this manuscript, but some discussion should be given to this.

Thank you for these comments. As noted, the *β-globin* 3' UTR confers significant stability to a transcript as well-documented in the literature (Volloch & Housman, 1981; Wilson & Deeley, 1995). We would point out that this is a standard control UTR utilised by many studies as a baseline, both within developmental biology and more broadly (examples: Hutchins et al., 2020; Shyu et al., 1989; Wiszniak et al., 2011). Identifying an alternative appropriate control is difficult given how little is known about transcript stability in this context (e.g., many transcripts may exhibit tissue-specific stability patterns). Our finding that the *Ciona twist1a-like* 3' UTR results in similar GFP expression to the contralateral *β-globin* control is a significant indicator that the substantial difference in fluorescence observed with vertebrate *Twist1* 3' UTRs is not simply a product of comparison to the *β-globin* control but truly represents a destabilising effect of these UTRs.

Regarding the tissue-specificity of *Twist1* transcript destabilisation, we agree that identifying upstream regulators specific to NCCs would be very interesting but falls outside the scope of this manuscript. Previous studies have revealed the differential expression of various RNA-Binding Proteins (RBPs) between premigratory and migratory CNCCs (Hutchins et al., 2022). It is also possible that tissue-specific differences in stability arise through post-translational modification of RBPs, which remains an unexplored mode of transcript regulation in neural crest development. We have added some text to the discussion to address these points, which can be found at lines **400-406**.

• The investigators examine mouse embryos and report that Twist1 mRNA but not protein is found in premigratory and early migratory CNCCs, but if I understand correctly, this is a different situation than chick which only has detectable Twist1 transcript in late migratory CNCCs. Given conservation of the Twist1 ARE, this is somewhat surprising. This significance of this apparent

difference (possibly relating to differences in NCC timing in mouse and chick?) might be discussed.

Thank you for noticing this difference – we were also intrigued by this apparent species difference.

Motivated by this question, we repeated our HCR staining for *Twist1* transcripts in chicken embryo sections, which we then imaged at higher magnification (40X), in line with the imaging performed in mouse embryo sections. While our previous observation that CNCCs only accumulate *Twist1* transcripts late in their migration into the pharyngeal arch holds true, this imaging revealed the presence of nuclear puncta throughout cells of the neural tube. Importantly, this observation is consistent with the activity of the *Twist1*+225kb enhancer that we observe throughout the neural tube. Considering these findings, the previously apparent difference between mouse and chicken does not hold. We are excited about these findings as they corroborate the *in vivo* significance of the *Twist1*+225kb enhancer in driving transcription of the endogenous *Twist1* gene. We have adjusted the wording of the text of the paper in a few places to reflect this finding and for consistency (e.g., from "*Twist1* is expressed by late migratory CNCCs" to "*Twist1* transcripts accumulate in late migratory CNCCs" in the abstract, lines **13-14**).

We have added additional figure panels (new Fig. 4f-g) and text to the manuscript addressing this, which can be found at lines **208-218**.

Minor points

• I could not open Supplemental Data tables 1-3 which indicated an invalid file format.

Apologies for the inconvenience, this arose due to file corruption during upload. We have replaced the tables with files that should be formatted correctly now.

• For in vivo enhancer experiments presented in Fig. 2c-h' I could not locate number of embryos that were scored. I was particularly curious for this information relating to the trunk neural tube vs. NCCs.

We have now updated the text to include the number of replicates for all experiments. For the trunk NCC electroporation experiment mentioned here, this result was obtained in 3/3 embryos (with 5/5 sections imaged and scored per embryo).

• In addition to TFAP2 and FOXD3, several other predicted regulators of the Twist1+225kb enhancer were predicted by FIMO, including HMGA1 were these tested by generation of modified enhancer constructs and found not to have an effect, or were these candidate regulators untested?

Thank you for this question. We decided to focus on a subset of candidate regulators that we had also shown to be co-expressed with *Twist1* by neural crest cells (TFAP2 and FOXD3). We also tested scrambling the MAF binding site, which did not cause a significant difference in activity (data not shown).

References

- Abitua, P. B., Wagner, E., Navarrete, I. A., and Levine, M. (2012). Identification of a rudimentary neural crest in a non-vertebrate chordate. *Nature* **492**:104-107.
- Bhattacharya, D., Rothstein, M., Azambuja, A.P., and Simoes-Costa, M. (2018). Control of neural crest multipotency by Wnt signaling and the Lin28/let-7 axis. *eLife* **7**:e40556.
- Bronner-Fraser, M., and Garcia-Castro, M. (2008). Manipulations of Neural Crest Cells or Their Migratory Pathways. *Methods in Cell Biology* **87**.
- Cohen, A. M., and Konisberg, I. R. (1975). A clonal approach to the problem of neural crest determination. *Developmental Biology* **46**(2):262-280.
- Hutchins, E. J., Piacentino, M. L., and Bronner, M. E. (2020). P-bodies are sites of rapid RNA decay during the neural crest epithelial-mesenchymal transition. *BioRxiv* doi: 10.1101/2020.07.31.231860.
- Hutchins, E. J., Gandhi, S., Chacon, J., Piacentino, M., and Bronner, M. E. (2022). RNA-binding protein Elavl1/HuR is required for maintenance of cranial neural crest specification. *eLife* **11**:e63600.
- Morrison, J. A., McLennan, R., Wolfe, L. A., Gogol, M. M., Meier, S., McKinney, M. C., Teddy, J. M., Holmes, L., Semerad, C. L., Box, A. C., Li, H., Hall, K. E., Perera, A. G., and Kulesa, P. M. (2017). Single-cell transcriptome analysis of avian neural crest migration reveals signatures of invasion and molecular transitions. *eLife* **6**:e28415.
- Shyu, A.-B., Greenberg, M. E., and Belasco, J. G. (1989). The *c-fos* transcript is targeted for rapid decay by two distinct mRNA degradation pathways. *Genes & Development* **3**:60-72.
- Simoes-Costa, M., Tan-Cabugao, J., Antoshechkin, I., Sauka-Spengler, T., and Bronner, M. E. (2014). Transcriptome analysis reveals novel players in the cranial neural crest gene regulatory network. *Genome Research* **24**:281-290.
- Todorov, L. G., Oonuma, K., Kusakabe, T. G., Levine, M. S. and Lemaire, L. A. (2024). Neural crest lineage in the protovertebrate model Ciona. *Nature* **635**:912-916.
- Volloch, V., and Housman, D. (1981). Stability of Globin mRNA in Terminally Differentiating Murine Erythroleukemia Cells. *Cell* **23**:509-514.
- Wilson, G. M., and Deeley, R. G. (1995). An Episomal Expression Vector System for Monitoring Sequence-Specific Effects on mRNA Stability in Human Cell Lines. *Plasmid* **33**:198-207.
- Wiszniak, S. E., Dredge, B. K., and Jensen, K. B. (2011). *HuB* (*elavl2*) mRNA is restricted to the germ cells by post-transcriptional mechanisms including stabilization of the message by DAZL. *PLoS ONE* **6**:e20773.
