# Supplementary material for: Ectomesenchymal identity emerges via relief of Twist1 transcript destabilisation
Source: Nat Commun. 2026 Aug 11;17:9642. doi: 10.1038/s41467-026-76557-6 (PMC13554066; doi:10.1038/s41467-026-76557-6)
Supplement: Supplementary file 3 [file 41467_2026_76557_MOESM3_ESM.pdf]

Description of Additional Supplementary Files

File Name: Supplementary Data 1
Description: Twist1 promoter region genomic contacts from Hi-ChIP data, obtained using DiffLoop. Reanalysis (galGal6) of data from Azambuja & Simoes-Costa, 2021. The Twist1 promoter is within the Chr2:29500034-29723798 region (shaded in grey). All contacts have an associated logFC (whole embryo vs. neural folds) and pvalue, as well as a log(counts per million) value. Statistical testing was performed by fitting an overdispersed Poisson regression with automatic correction for size factors, and correction for multiple testing (represented by the False Discovery Rate, FDR).

File Name: Supplementary Dsata 2
Description: Putative Twist1 enhancer regions tested in this study.

File Name: Supplementary Data 3
Description: Relevant DNA sequences.
