# Supplementary material for: Ectomesenchymal identity emerges via relief of Twist1 transcript destabilisation
Source: Nat Commun. 2026 Aug 11;17:9642. doi: 10.1038/s41467-026-76557-6 (PMC13554066; doi:10.1038/s41467-026-76557-6)
Supplement: Supplementary file 4 [file 41467_2026_76557_MOESM7_ESM.pdf]

nature portfolio

Corresponding author(s): Megan Martik

Last updated by author(s): Jun 5, 2026

Reporting Summary

Nature Portfolio wishes to improve the reproducibility of the work that we publish. This form provides structure for consistency and transparency in reporting. For further information on Nature Portfolio policies, see our Editorial Policies and the Editorial Policy Checklist.

Statistics

For all statistical analyses, confirm that the following items are present in the figure legend, table legend, main text, or Methods section.

n/a	Confirmed	
☐	☒	The exact sample size (n) for each experimental group/condition, given as a discrete number and unit of measurement
☐	☒	A statement on whether measurements were taken from distinct samples or whether the same sample was measured repeatedly
☐	☒	The statistical test(s) used AND whether they are one- or two-sided<br>*Only common tests should be described solely by name; describe more complex techniques in the Methods section.*
☒	☐	A description of all covariates tested
☐	☒	A description of any assumptions or corrections, such as tests of normality and adjustment for multiple comparisons
☐	☒	A full description of the statistical parameters including central tendency (e.g. means) or other basic estimates (e.g. regression coefficient) AND variation (e.g. standard deviation) or associated estimates of uncertainty (e.g. confidence intervals)
☐	☒	For null hypothesis testing, the test statistic (e.g. F, t, r) with confidence intervals, effect sizes, degrees of freedom and P value noted<br>*Give P values as exact values whenever suitable.*
☒	☐	For Bayesian analysis, information on the choice of priors and Markov chain Monte Carlo settings
☒	☐	For hierarchical and complex designs, identification of the appropriate level for tests and full reporting of outcomes
☒	☐	Estimates of effect sizes (e.g. Cohen's d, Pearson's r), indicating how they were calculated

Our web collection on statistics for biologists contains articles on many of the points above.

Software and code

Policy information about availability of computer code

Data collection	Images were collected using Leica LAS-X (Leica M205 stereoscope) and Zeiss ZEN (Zeiss 780 LSM upright confocal microscope).
Data analysis	Previously published Hi-ChIP data (Azambuja & Simoes-Costa, 2021) was analysed using the following softwares referenced in the methods section: HiC-Pro, Hichipper, Diffloop and Sushi. Previously published H3K27ac and TFAP2A and TFAP2B CUT&RUN datasets (Bhattacharya et al., 2020; Rothstein & Simoes-Costa, 2020) were analysed usingthe following softwares as referenced in the methods section: Cutadapt, Bowtie2, samtools, and deepTools. Previously published ATAC-Seq datasets (Hovland et al., 2022) were analysed with the following softwares referenced in the methods section: Cutadapt, Bowtie2, samtools, and deepTools. To visualise ATAC and Cut & Run datasets together over a chromosomal interval of interest, the package PlotGardener in R was utilised. Transcription factor binding site analysis was performed using the FIMO tool in MEME Suite. The previously published single cell RNA-sequencing dataset (Morrison et al., 2017) was analysed as follows: reads were mapped to the genome using STAR, then individual cell .bam files were compiled into a single counts table using featureCounts. Scanpy was used to process data in python. Image analysis was performed using FIJI, RStudio (ggplot2) and Graphpad PRISM.

For manuscripts utilizing custom algorithms or software that are central to the research but not yet described in published literature, software must be made available to editors and reviewers. We strongly encourage code deposition in a community repository (e.g. GitHub). See the Nature Portfolio guidelines for submitting code & software for further information.

Data

Policy information about availability of data

All manuscripts must include a data availability statement. This statement should provide the following information, where applicable:

- Accession codes, unique identifiers, or web links for publicly available datasets
- A description of any restrictions on data availability
- For clinical datasets or third party data, please ensure that the statement adheres to our policy

Raw image files are available at the following link: https://figshare.com/s/38f7fd544c8b7899aa03.

Research involving human participants, their data, or biological material

Policy information about studies with human participants or human data. See also policy information about sex, gender (identity/presentation), and sexual orientation and race, ethnicity and racism.

Reporting on sex and gender	N/A
Reporting on race, ethnicity, or other socially relevant groupings	N/A
Population characteristics	N/A
Recruitment	N/A
Ethics oversight	N/A

Note that full information on the approval of the study protocol must also be provided in the manuscript.

Field-specific reporting

Please select the one below that is the best fit for your research. If you are not sure, read the appropriate sections before making your selection.

☒ Life sciences ☐ Behavioural & social sciences ☐ Ecological, evolutionary & environmental sciences

For a reference copy of the document with all sections, see nature.com/documents/nr-reporting-summary-flat.pdf

Life sciences study design

All studies must disclose on these points even when the disclosure is negative.

Sample size	Sample sizes were selected in accordance with standards in developmental biology. All n's are included in the figure legends of the manuscript.
Data exclusions	No data were excluded.
Replication	Reproducibility was confirmed by confirming each result in at least three independent embryos for all experiments. All attempts at replication were successful.
Randomization	Embryos were randomly assigned to each experimental group.
Blinding	N/A

Reporting for specific materials, systems and methods

We require information from authors about some types of materials, experimental systems and methods used in many studies. Here, indicate whether each material, system or method listed is relevant to your study. If you are not sure if a list item applies to your research, read the appropriate section before selecting a response.

Materials & experimental systems

n/a	Involved in the study	
☐	☒	Antibodies
☒	☐	Eukaryotic cell lines
☒	☐	Palaeontology and archaeology
☐	☒	Animals and other organisms
☒	☐	Clinical data
☒	☐	Dual use research of concern
☒	☐	Plants

Methods

n/a	Involved in the study	
☒	☐	ChIP-seq
☒	☐	Flow cytometry
☒	☐	MRI-based neuroimaging

Antibodies

Antibodies used

Primary antibodies: 1:50 mouse anti-TWIST1 (abcam ab50887, lot number 1083809), 1:200 goat anti-SOX10 (R&D AF2864, lot number VRY1423081), 1:900 rabbit anti-SOX2 (abcam ab97959, lot number 1131344-4), and 1:200 goat anti-GFP (Rockland 600-101-215, lot number 51370). Secondary antibodies (all at 1:200): donkey anti-mouse Alexa 647 (Invitrogen A31571, lot number 3141854), donkey anti-goat 568 (Invitrogen A11057, lot number 2776028), donkey anti-goat 488 (Invitrogen A11055, lot number 2747580), and donkey anti-rabbit 594 (Invitrogen A21207, lot number 3148833).

Validation

Validation statements for all antibodies can be found on the manufacturer websites via the links below:
Mouse anti-TWIST1 (abcam ab50887) - https://www.abcam.com/en-us/products/primary-antibodies/twist-antibody-twist2c1a-ab50887
Goat anti-SOX10 (R&D AF2864) - https://www.rndsystems.com/products/human-sox10-antibody_af2864
Rabbit anti-SOX2 (abcam ab97959) - https://www.abcam.com/en-us/products/primary-antibodies/sox2-antibody-ab97959
Goat anti-GFP (Rockland 600-101-215) - https://www.rockland.com/categories/primary-antibodies/gfp-antibody-600-101-215/?srsltid=AfmBOoqq-DdsTop2mFQod53TdIC5Lv5Lrlo7XRX3QsAw7Os60_M_aPqE
Donkey anti-mouse Alexa 647 (Invitrogen A31571) - https://www.thermofisher.com/antibody/product/Donkey-anti-Mouse-IgG-H-L-Highly-Cross-Adsorbed-Secondary-Antibody-Polyclonal/A-31571
Donkey anti-goat 568 (Invitrogen A11057) - https://www.thermofisher.com/antibody/product/Donkey-anti-Goat-IgG-H-L-Cross-Adsorbed-Secondary-Antibody-Polyclonal/A-11057
Donkey anti-goat 488 (Invitrogen A11055) - https://www.thermofisher.com/antibody/product/Donkey-anti-Goat-IgG-H-L-Cross-Adsorbed-Secondary-Antibody-Polyclonal/A-11055
Donkey anti-rabbit 594 (Invitrogen A21207) - https://www.thermofisher.com/antibody/product/Donkey-anti-Rabbit-IgG-H-L-Highly-Cross-Adsorbed-Secondary-Antibody-Polyclonal/A-21207

Animals and other research organisms

Policy information about studies involving animals; ARRIVE guidelines recommended for reporting animal research, and Sex and Gender in Research

Laboratory animals

Wild-type fertile Gallus gallus (chicken) eggs were obtained from a local farm (Petaluma Farms, CA) then incubated at 37 degrees C to obtain embryos of the desired stage. Wild-type AB strain Danio rerio (zebrafish) were maintained in a dedicated facility and crossed to produce embryos. Wildtype C57Bl/6J mice (Jackson Laboratories) were maintained in a dedicated facility (22 degrees C, 40-60% RH) with standard inbreeding schemes and housed on a 12/12h light/dark cycle with ad libitum access to food and water.

Wild animals

N/A

Reporting on sex

All experiments were performed on a mixture of male and female embryonic samples; no sex-based analysis was performed.

Field-collected samples

N/A

Ethics oversight

All animal work was carried out in full compliance with approved protocols (UC Berkeley AUP-2021-03-14107-1 and AUP-2017-12-10556-2, Stanford University APLAC institutional guidelines set by the VSC).

Note that full information on the approval of the study protocol must also be provided in the manuscript.

Plants

Seed stocks	N/A
Novel plant genotypes	N/A
Authentication	N/A
